# Regulation of volatile and non-volatile pheromone attractants depends upon male social status

**DOI:** 10.1038/s41598-018-36887-y

**Published:** 2019-01-24

**Authors:** M. Thoß, K. C. Luzynski, V. M. Enk, E. Razzazi-Fazeli, J. Kwak, I. Ortner, D. J. Penn

**Affiliations:** 1 Department of Integrative Biology and Evolution, Konrad Lorenz Institute of Ethology, University of Veterinary Medicine, Vienna, Austria; 2 Proteomics Unit, VetCORE Facility for Research, University of Veterinary Medicine, Vienna, Austria; 3 Department of Integrative Biology and Evolution, Research Institute of Wildlife Ecology, University of Veterinary Medicine, Vienna, Austria; 4 Institute of Statistics and Mathematical Methods in Economics, TU Wien, Vienna, Austria; 5 Present Address: International Flavors & Fragrances Inc., Union Beach, New Jersey, USA; 6 Present Address: Department of Mathematics, Statistics Section, KU Leuven, Leuven, Belgium

## Abstract

We investigated the regulation of chemical signals of house mice living in seminatural social conditions. We found that male mice more than doubled the excretion of major urinary proteins (MUPs) after they acquired a territory and become socially dominant. MUPs bind and stabilize the release of volatile pheromone ligands, and some MUPs exhibit pheromonal properties themselves. We conducted olfactory assays and found that female mice were more attracted to the scent of dominant than subordinate males when they were in estrus. Yet, when male status was controlled, females were not attracted to urine with high MUP concentration, despite being comparable to levels of dominant males. To determine which compounds influence female attraction, we conducted additional analyses and found that dominant males differentially upregulated the excretion of particular MUPs, including the pheromone MUP20 (darcin), and a volatile pheromone that influences female reproductive physiology and behavior. Our findings show that once male house mice become territorial and socially dominant, they upregulate the amount and types of excreted MUPs, which increases the intensities of volatiles and the attractiveness of their urinary scent to sexually receptive females.

## Introduction

House mice (*Mus musculu*s) excrete large quantities of major urinary proteins (MUPs) that bind and transport hydrophobic ligands, including several volatile pheromones^1–3^. Upon excretion, MUPs slow down the release of volatiles from scent marks^4–8^, which may prolong their attraction and influence on conspecifics. MUPs are often suggested to show high individual diversity and thereby mediate individual and kin recognition^9,10^. They are encoded by 21 paralogous loci, but MUP genes are highly homologous^11,12^ and no individual variation has been detected in wild populations^13^. Interestingly, MUP excretion is sexually dimorphic^5,14,15^, under endocrine control^16–18^, and dynamically regulated^19^ depending upon health^20–22^, nutritional status^23^, and social interactions^24,25^. Our aims here were to test whether house mice regulate the excretion of MUPs or volatile pheromone ligands depending upon their social status, and whether such regulation influences the attractiveness of their odor to potential mates^24^.

Male house mice are territorial and much evidence indicates that intra- and inter-sexual selection are mediated by chemosensory signals. Dominant territorial males scent mark their territories, and males deposit more scent-marks after winning an agonistic encounter compared to losers (winners are commonly labeled as ‘dominants’ and losers as ‘subordinates’)^26–28^. Females are attracted to male urinary scent^29–31^ and especially to the scent of ‘dominant’ males^32–35^. Male reproductive success correlates with scent-marking when male social status is controlled and females are free to select their mates^36^. Male urine also has priming effects on female reproductive physiology (accelerating puberty, synchronizing estrus, and blocking pregnancy^37^), and especially if males are socially ‘dominant’^38^. Several volatile odor compounds (VOCs) have been identified as sexual pheromones (eliciting sexual attraction, priming effects, or both), including α- and β- farnesene^39^, 2-*sec-*Butyl-4, 5-dihydrothiazole (SBT), 3, 4-dehydro-exo-brevicomin (DHB), and 6-hydroxy-6-methyl-3-heptanone (HMH^40^), though not in all strains^41^. Exposure to a combination of male pheromones (SBT, DHB, and HMH) induces female olfactory preferences for these compounds^40^ and accelerates vaginal opening^42^. ‘Dominant’ males excrete higher quantities of MUPs^24,43–45^ and volatile pheromone ligands (α- and β- farnesene and SBT^39,46^) than defeated ‘subordinates’. These findings might help explain how females discriminate winners from losers^32,34,35^ and how dominant, territorial males achieve greater reproductive success than subordinates^47–49^.

These studies have provided valuable insights into chemosensory-mediated sexual selection, however, several questions still need to be addressed. First, studies on chemical communication in mice are primarily conducted with laboratory strains (*Mus laboratorius*^50^), but their ecological relevance should not be assumed^51,52^ (especially since male laboratory mice are not territorial and most strains are not as aggressive as wild mice^53,54^). Second, previous studies have mainly used the outcome of short-term, dyadic agonistic interactions as proxies for social status, and winners and losers are labeled as ‘dominants’ and ‘subordinates’, respectfully. These assays are also used to study social defeat^55–57^, which results in behavioral^58^ and physiological changes^55,57^, including detrimental health effects^59^. However, they do not necessarily provide a valid proxy for assessing a male’s ability to obtain a territory nor subsequent changes in behavior and physiology^57,59,60^ of dominants or subordinates under more natural social conditions. Indeed, a recent study on laboratory mice indicates that winning dyadic agonistic assays did not predict social dominance in more complex social environments^45^. Third, it is unclear whether MUP excretion is regulated according to social status, or vice versa, as previously suggested^44^ and it is also unclear whether winners upregulate or losers down-regulate MUPs and volatile pheromones. Longitudinal studies are needed to examine pheromone excretion in social interactions and with controls for comparison. Finally, the regulation of MUPs and volatiles have mainly been investigated independently, with few exceptions^61^ and studies are needed to examine how MUP regulation influences odor, as well as the excretion of volatiles.

We conducted a study on social behavior and regulation of MUPs and other pheromones in wild-derived (F1 from wild-caught) house mice (*Mus musculus musculus)* to address the following aims: (1) We tested whether MUP production provides a reliable indicator of social status in seminatural conditions over 12 weeks, and we also analyzed protein excretion from controls kept in standard housing conditions during the same time. (2) We conducted olfactory assays to test whether female mice discriminate the scent of dominant versus subordinate males^32^ and whether variation in male urinary protein concentration influences female preferences when male social status was controlled. (3) We conducted in-depth proteomic analyses using SWATH (Sequential Window Acquisition of All Theoretical Fragment Ion Mass Spectra) to measure the regulation of specific MUP proteoforms, and gas chromatography- mass spectrometry (GC-MS) to analyze variation in specific volatile pheromones. We expected dominant males to upregulate, or subordinates to down-regulate (social defeat) protein excretion, or both. To determine whether regulation is temporary or long-lasting, we continued measuring MUP excretion after social interactions were terminated. We expected females to have lower levels of protein excretion than males, and to regulate protein excretion depending upon their social status, as previously suggested^62^. We expected that females would discriminate and prefer the urine of dominant over that of subordinate males, and that they would prefer the scent of males with high MUP concentration when social status is controlled (as observed in rats^63^). Finally, we expected that mice would show differential regulation of particular MUP proteoforms^43^, volatile pheromones^39^, or both, depending upon their social status.

## Results

### MUP regulation

**Figure 1:**
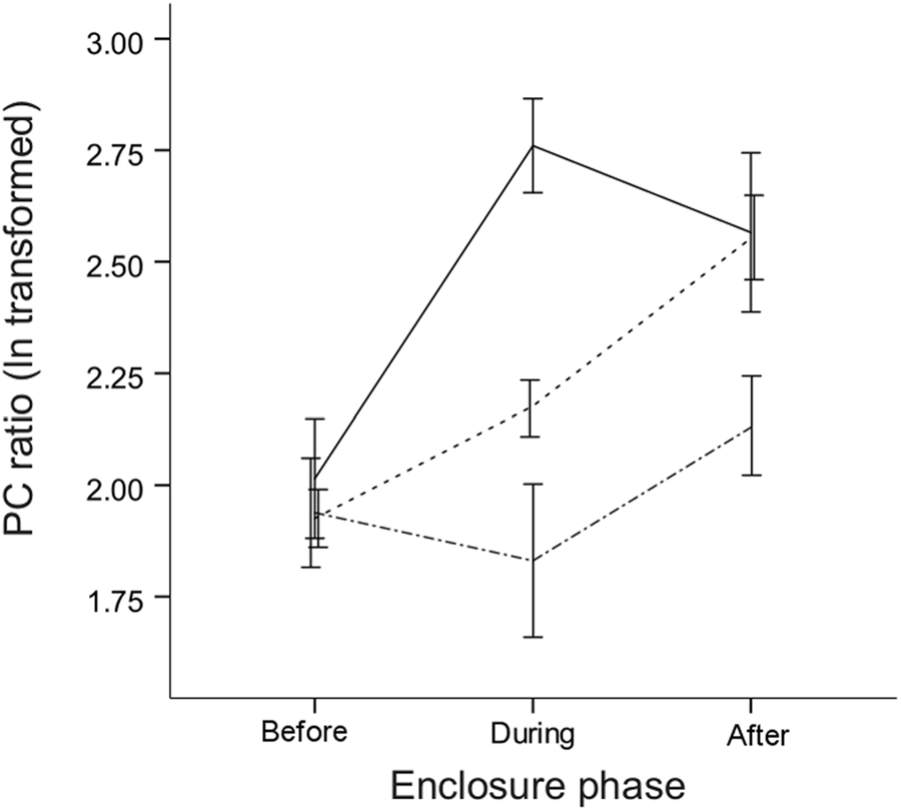
Dominant males upregulate urinary protein excretion in seminatural conditions. Male urinary protein excretion (PC ratio) before, during, and after housing in seminatural enclosures for socially dominant (solid line) and subordinate (dot-dashed line) males compared to caged control males (dashed line). Mice were repeatedly sampled before (two urine collections) and during enclosure phase (three urine collections). Error bars indicate mean ± 1 s.e.m. (standard error of the mean).

Males that became socially dominant (DOM) in the seminatural enclosures upregulated urinary protein excretion, as expected, whereas subordinates (SUB) and controls (CTRL) showed no significant changes during this time (Fig. 1). Linear mixed-effects (LME) modelling of urinary protein excretion (log transformed PC ratio) showed a significant interaction between male social status and enclosure phase (social status * enclosure phase: *F*_*4*,*245*_ = 4.3, p* = *0.002; Supplementary Table S4). Tukey *post hoc* analyses revealed that males significantly increased urinary protein excretion upon becoming socially dominant (∆_during-before_ = 0.80 ± 0.21 (mean PC ratio ± s.e.m.), Z = 3.70, p < 0.01; Supplementary Table S4), and thus, dominant males excreted more urinary protein than subordinates (DOM: 2.79 ± 1.11 vs SUB: 1.83 ± 0.17, Z = 3.7, p < 0.01) and caged controls (DOM: 2.79 ± 1.11 vs CTRL: 2.17 ± 0.06, Z = 3.9, p < 0.01; Supplementary Table S4) during the enclosure phase. Subordinate males did not down-regulate urinary protein excretion and had levels similar to caged control males (SUB: 1.83 ± 0.17 vs CTRL: 2.17 ± 0.06, Z = −1.2, p = 0.95; Supplementary Table S4). Four weeks after removing the mice from the enclosures, male urinary protein excretion no longer differed between dominant males versus subordinates or controls (all p > 0.9). Unlike social status, age was not associated with urinary protein excretion (*F*_*1*,*245*_ = 0.3, p* = *0.60; Supplementary Table S4).

**Figure 2:**
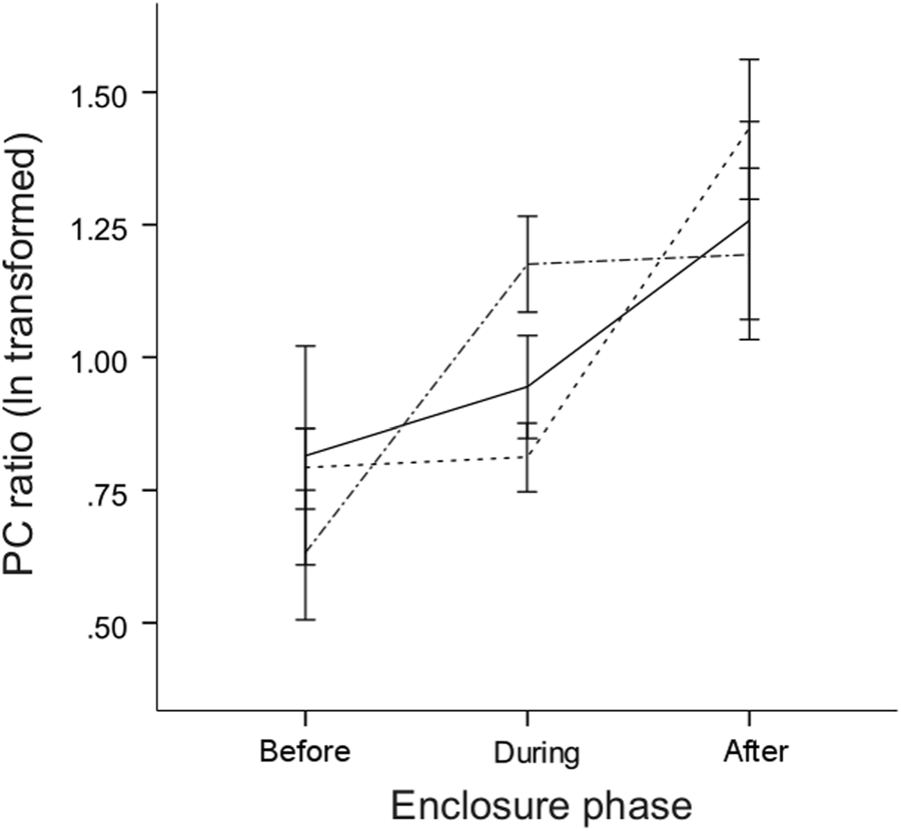
Female urinary protein excretion increases over time. Female urinary protein excretion (PC ratio) before, during, and after housing in seminatural enclosures for socially dominant (solid line) and subordinate (dot-dashed line) females compared to caged control females (dashed line). Mice were repeatedly sampled before (two collections) and during (three urine collections) the enclosure phases. Error bars indicate mean ± 1 s.e.m.

Unlike males, female mice did not regulate their urinary protein excretion according to social status (Fig. 2). LME modelling of female PC ratio showed a significant interaction of social status and enclosure phase (social status * enclosure phase: *F*_*4*,*288*_ = 3.5, p < 0.01; Supplementary Table S5). Tukey *post hoc* analyses revealed a significant increase in urinary protein excretion in subordinate females during the enclosure phase compared to levels before (Δ_during-before_ = 0.51 ± 0.13, Z = 4.02, p < 0.01; Supplementary Table S5); however, there was no difference in urinary protein excretion between dominant, subordinate, and caged control females during the enclosure phase (all p > 0.22).

#### Total urinary protein excretion

LME modelling of total (unadjusted) urinary protein excretion in males showed a significant interaction of male social status and enclosure phase (social status * enclosure phase: *F*_*4*,*245*_ = 8.8, p < 0.001) with dominant and subordinate mice increasing excretion during the enclosure phase compared to prior levels (DOM ∆_during-before_ = 1.63 ± 0.23 mg ml^−1^, Z = 7.2, p < 0.001; SUB ∆_during-before_ = 1.95 ± 0.24 mg ml^−1^, Z = 8.0, p < 0.001; Supplementary Table S4). However, no difference in total urinary protein excretion was observed between social status groups during the enclosures (all p > 0.09; Supplementary Fig. S3, Table S4). Male mice showed a significant increase in total urinary protein excretion over time (*F*_*2*,*245*_ = 30.6, p < 0.01; DOM ∆_after-before_ = 1.66 ± 0.30 mg ml^−1^, Z = 5.5, p < 0.001; SUB ∆_after-before_ = 2.48 ± 0.38 mg ml^−1^, Z = 6.6, p < 0.001; CTRL ∆_after-before_ = 1.92 ± 0.20 mg ml^−1^, Z = 6.6, p < 0.001; Supplementary Fig. S3, Table S4). Before the enclosure phase, total urinary protein excretion was greater for caged controls than for males that later became subordinate (∆_SUB-CTRL_ = −0.70 ± 0.20 mg ml^−1^, Z = −3.6, p = 0.009; Supplementary Table S4), though this difference disappeared during and after the enclosure phase (all p > 0.14). Total urinary protein excretion was not associated with male body mass (*F*_*4*,*245*_ = 0.01, p = 0.91). Female mice differed in total urinary protein excretion during the enclosure phase with dominant and subordinate females excreting more total urinary protein than caged controls (social status * enclosure phase: *F*_*4*,*288*_ = 10.4, p < 0.01; ∆_DOM-CTRL_ = 0.25 ± 0.06 mg ml^−1^, Z = 3.8, p < 0.01; ∆_SUB-CTRL_ = 0.28 ± 0.05 mg ml^−1^, Z = 5.6, p < 0.01; Supplementary Fig. S4, Table S5). Total protein excretion levels did not differ between social status groups before or after the enclosure phase (all p > 0.88).

#### Creatinine excretion

Subordinate males significantly increased creatinine excretion during the enclosure phase compared to previous levels (social status * enclosure phase: *F*_*4*,*245*_ = 2.7, p = 0.03; SUB ∆_during-before_ = 0.73 ± 0.22, Z = 3.40, p = 0.02; Supplementary Table S4), whereas dominant males and caged controls did not change creatinine excretion (DOM ∆_during-before_ = −0.11 ± 0.18, Z = −0.61, p* = *0.99; CTRL ∆_during-before_ = 0.02 ± 0.10, Z = 0.22, p = 1.00; Supplementary Table S4). There was no relationship between creatinine excretion and any of the fixed effects for female house mice (all p > 0.15; Supplementary Table S5). Female social status groups did not differ in creatinine levels at any time during the study (all p > 0.18).

#### Body mass and social status

There was no effect of initial mass on male or female social status (male: *U* = 119, p = 0.85; females: *U* = 104, p = 0.55; Supplementary Table S6). Mean mass and change in mass during the enclosure period were not significantly different between dominant and subordinate males (all p > 0.50).

#### Sex differences and housing conditions (enclosure v. caged control)

PC ratio, total urinary protein, and creatinine excretion were significantly greater for males than females, regardless of housing conditions (all p < 0.001; Supplementary Table S7). However, mice in seminatural conditions had a higher PC ratio (Χ² = 5.2, p = 0.02) and total urinary protein level (Χ² = 12.5, p < 0.001) compared to caged controls, yet there was no effect of housing on creatinine (Χ² = 1.1, p = 0.30). Interestingly, the sex * housing interaction had a significant effect on total urinary protein excretion (Χ² = 28.1, p < 0.001), indicating that sex difference in protein excretion was greater for the caged controls (M:F ratio = 6.61; Supplementary Table S7) than for enclosure mice (M:F ratio = 4.96).

### Olfactory discrimination

**Figure 3:**
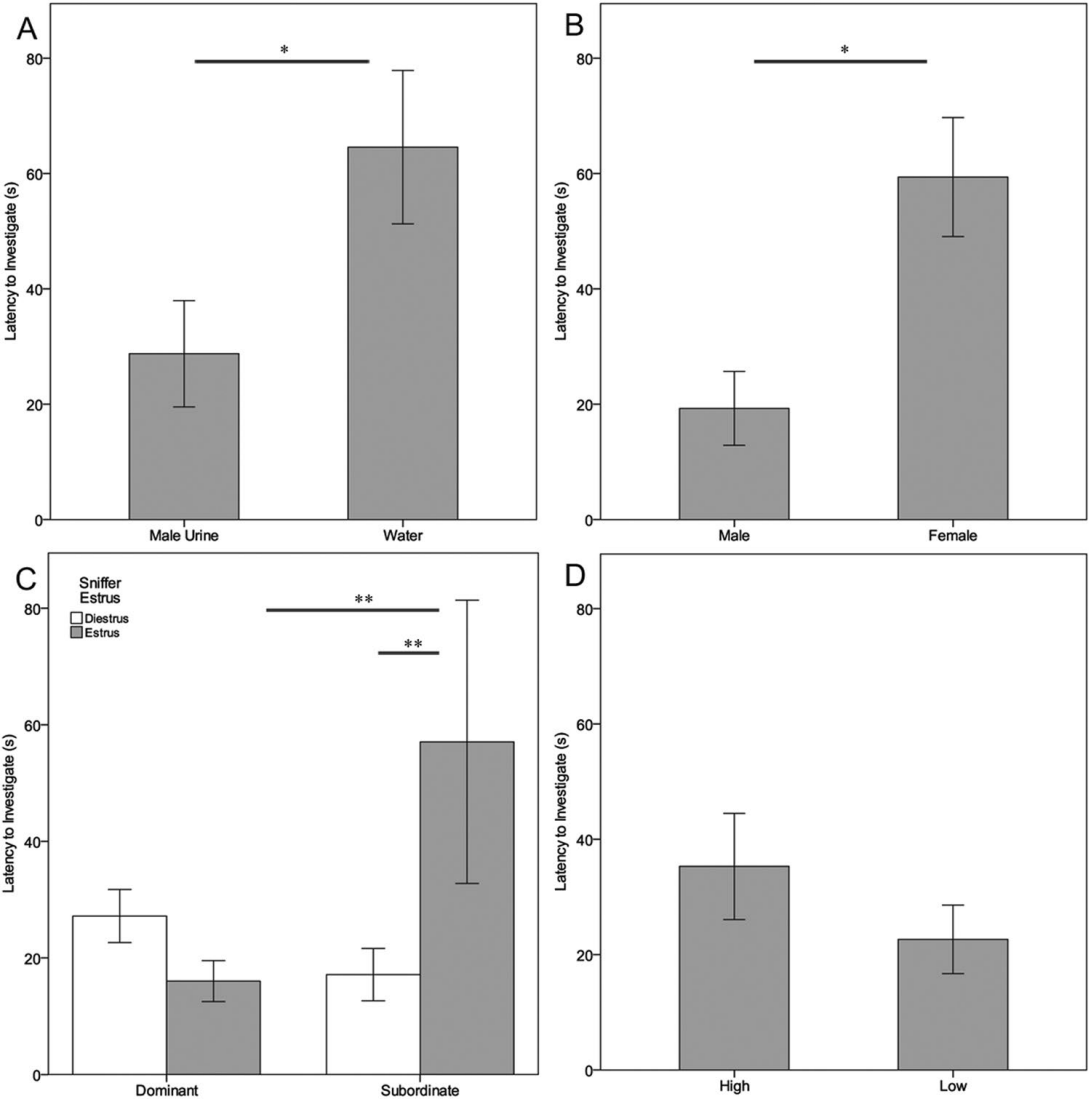
Female olfactory preferences measured by latency to investigate two different urinary stimuli in a Y-maze apparatus: (**A**) adult male urine versus water (N = 15); (**B**) adult male versus adult female urine (N = 10); (**C**) dominant versus subordinate male urine (N = 10, tested in diestrus (white bars) and estrus (gray bars); and (**D**) high versus low urinary protein concentration of control males (N = 10, 3:1 PC ratio). Significance of p < 0.05 indicated by ‘*’, p < 0.01 indicated by ‘**’. Error bars indicate ± 1 s.e.m.

In our olfactory assays, we confirmed that estrous females showed a significant initial attraction toward male urine over a neutral (water) stimulus (first entry: 12 urine vs 3 water, p = 0.04; latency to visit: ∆_urine-water_ = −44.7 ± 16.6 s, p = 0.04; Fig. 3A, Supplementary Table S8), and for male over female urine (first entry: 9 male vs 1 female, p = 0.02; latency to visit: ∆_male-female_ = −40.1 ± 10.2 s, p = 0.04; Fig. 3B, Supplementary Table S8). Estrous females showed a significant initial preference for the scent of dominant males over subordinates (first entry: 9 DOM vs 1 SUB, p = 0.02; latency to visit: ∆_DOM-SUB_ = −41.0 ± 1.7 s, p = 0.02; Fig. 3C, Supplementary Table S9), whereas diestrous females showed no significant preferences (first entry: 3 DOM vs 7 SUB, p = 0.12; latency to visit: ∆_DOM-SUB_ = 10.1 ± 5.1 s, p = 0.31). Contrary to our expectations, females showed no initial preferences for male urine with high versus low protein concentration (first entry: 5 vs 5, p = 0.25; latency to visit: ∆_high-low_ = 12.7 ± 11.8 s, p = 0.36; Fig. 3D, Supplementary Table S8).

To determine how females discriminate dominant from subordinate males, we conducted analyses to examine how social status influenced the excretion of particular MUP proteoforms and volatile pheromones, using high-resolution proteomics and GC-MS analyses, respectfully.

### Social status and specific MUPs (SWATH proteomic analysis)

#### Predicting social status from urinary protein before enclosure phase

Graphical and multivariate statistical analyses indicated significant differences in the relative proportions of MUP4, MUP5 and MUP17 between dominant and subordinate males before the enclosure phase (ANOSIM: global R = 0.23, p = 0.057; Supplementary Fig. S5, Supplementary Table S10). However, after correcting for multiple testing, these differences were no longer significant (Benjamini-Hochberg correction, all p ≥ 0.09). There were no differences in protein amount of MUPs (ANOSIM: global R = −0.01, p = 0.49), relative proportions of non-MUP proteins (ANOSIM: global R = −0.13, p = 0.90) or protein amount of non-MUP proteins (ANOSIM: global R = −0.03, p = 0.53) between dominant and subordinate males before the enclosure phase (Supplementary Fig. S5, Table S2 models 1–4).

#### Effect of social status on urinary protein during enclosure phase

Graphical and multivariate statistical analyses indicated significant differences in the relative proportions of three non-MUPs between dominant and subordinate males during the enclosure phase (ANOSIM: global R = 0.34, p = 0.018; Supplementary Fig. S6). However, after Benjamini-Hochberg correction for multiple testing, those differences are not significant (all p ≥ 0.95; Supplementary Table S10). There were no differences between dominant and subordinate males in relative proportions of MUPs (ANOSIM: global R = 0.17, p = 0.13; Supplementary Fig. S6), in the amount of MUP protein (ANOSIM: global R = 0.17, p = 0.15), nor in the amount of non-MUP proteins (ANOSIM: global R = 0.20, p = 0.10) during the experiment (Supplementary Table S2 models 5–8).

#### Effect of housing conditions on urinary protein excretion

Examining within-individual changes due to seminatural housing conditions showed significant effects on the amount (ANOSIM: global R = 0.35, p < 0.001), but not relative intensity of MUP protein excreted (ANOSIM: global R = 0.04, p = 0.11; Supplementary Fig. S7). Subsequent univariate tests revealed significant increases in protein amount of all measured MUPs, except MUP3 (see Supplementary Table S10). Seminatural conditions also affected the relative intensity of 26 non-MUP proteins (ANOSIM: global R = 0.230, p < 0.001; Supplementary Table S10) and the amount of all but 10 non-MUP proteins (ANOSIM: global R = 0.638, p < 0.0010; Supplementary Fig. S7, Table S10 and Table S2 models 9–12).

#### Fold change in urinary protein production

Fold change in total urinary protein excretion was highly correlated with fold change in MUP excretion overall (ρ = 0.91, R² = 0.85, p < 0.001), and separately for MUP1, MUP2, MUP3, MUP5, MUP17, and MUP20 (all ρ ≥ 0.64, all p ≤ 0.011) but not MUP4 (ρ = 0.28, p = 0.28). Dominant and subordinate males did not differ in the extent of fold change in relative intensity or protein amount of urinary proteins (MUP relative intensity, ANOSIM: global R = −0.02, p = 0.49; MUP protein amount, ANOSIM: global R = −0.05, p = 0.58; non-MUP relative intensity, ANOSIM: global R = 0.06, p = 0.19; non-MUP protein amount, ANOSIM: global R = 0.04, p = 0.25; Supplementary Fig. S8). However, when fold change in urinary proteins was compared to the null expectation (no change with social experience), the amount of MUP2, MUP5, MUP17, and MUP20 were significantly upregulated in the urine of dominant males (Supplementary Table S11). Surprisingly, the relative intensity of MUP5 showed a negative fold change in dominant males’ urine. On the contrary, fold changes in protein amount and relative intensity of MUPs in subordinate males did not differ from the null expectation.

### Social status and MUP-bound pheromones and other volatiles

**Figure 4:**
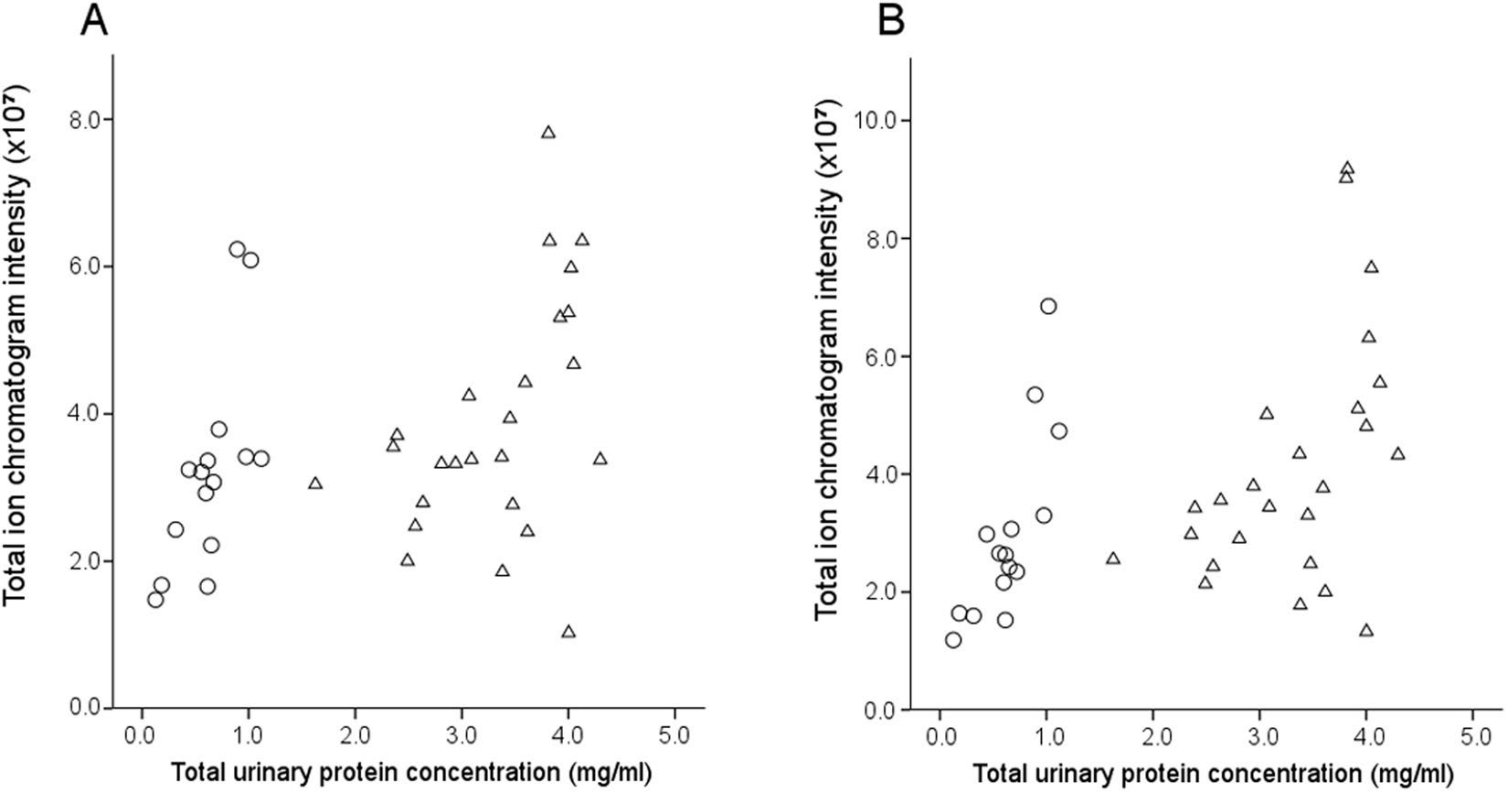
Volatile intensity is correlated with total urinary protein concentration. Correlation of total ion chromatogram intensity (measured by GC-MS) and total urinary protein concentration in (**A**) intact and (**B**) denatured urine samples of females (circles) and males (triangles). Panels (A,B) have different y-axes.

We found a strong correlation between total urinary protein excretion and the total ion chromatogram intensity of all peaks detected and analyzed (from GC-MS) for both intact and denatured urine (Pearson correlation, N = 40, intact urine: ρ = 0.45, p = 0.003; Fig. 4A; denatured urine: ρ = 0.54, p < 0.001; Fig. 4B). We examined whether social status influenced the intensity of pheromones that are MUP ligands (targeted compound approach), and found significant grouping according to social status among males (ANOSIM, intact urine: global R = 0.43, p = 0.009; denatured urine: global R = 0.51, p = 0.002; Supplementary Fig. S9), but not females (ANOSIM, intact urine: global R = −0.02, p = 0.50; denatured urine: global R = −0.12, p = 0.65). SPLS-DA and univariate tests showed that dominant males released more HMH compared to subordinates from both intact and denatured urine samples (intact urine: p = 0.007; denatured urine: p = 0.003). However, there was no compound that differentiated dominant from subordinate females, neither in intact nor in denatured urine (all p ≥ 0.11). We re-ran the analysis using the exploratory data set, and again we found significant grouping according to social status for males (ANOSIM, intact urine: global R = 0.54, p = 0.002; denatured urine: global R = 0.62, p = 0.001; Supplementary Fig. S10), but not for females (intact urine: global R = 0.11, p = 0.13; denatured urine: global R = −0.02, p = 0.55). However, subsequent SPLS-DA and univariate tests showed that dominant and subordinate males had no discernible volatile profiles in either the intact or denatured urine samples (all p ≥ 0.09). Similarly, dominant and subordinate females did not differ significantly in any compound released from intact or denatured urine samples (all p ≥ 0.13).

We re-ran the analysis with the targeted approach data set and found significant grouping according to housing conditions of males (ANOSIM, intact urine: global R = 0.24, p = 0.008; denatured urine: global R = 0.38, p = 0.001), but not females (all p ≥ 0.07). SPLS-DA and univariate tests showed that caged control males’ urine released significantly more SBT than urine of enclosure males (intact urine: DOM vs CTRL: p = 0.007, SUB vs CTRL: p = 0.006, DOM vs SUB: p = 1; denatured urine: DOM vs CTRL: p = 0.023, SUB vs CTRL: p = 0.03, DOM vs SUB: p = 1; Supplementary Fig. S11).

## Discussion

Our findings show that male house mice upregulated urinary protein excretion once they acquired a territory and became socially dominant. Females showed more attraction to the scent of dominant over subordinate males, though only during estrus^32^. Contrary to our expectations, male urinary protein concentration had no influence on female preferences when male social status was controlled. This finding suggests that female attraction to dominant males was due to changes in the urine other than the amount of urinary protein. We found that dominant males upregulated particular MUP proteoforms, including MUP20, a sexual attractant, and HMH, a volatile male pheromone. MUP excretion was correlated with an overall intensity of the VOCs in urine, and MUP upregulation may therefore increase and prolong the volatile signals in male scent marks. These findings show that territorial male mice upregulate the excretion of both volatile and non-volatile urinary chemical compounds that attract and influence the reproductive behavior of female mice^42,64–66^. Below we address our main results in more detail.

We found that males upregulated MUP excretion after acquiring a territory, which is consistent with previous studies^24,43–45^ and our results provide several additional findings. First, the differences in MUP excretion between dominant and subordinate males emerged after and not before social status was established. Interestingly, this finding is consistent with growing evidence that social behavior can affect gene expression^67^, which contrasts with the usual assumption that gene expression can influence behavior, but not vice versa. Second, subordinate males did not significantly decrease urinary protein excretion, contrary to what we expected from laboratory studies on social defeat. This result is also surprising given that losers of agonistic encounters reduce scent marking^26^ and males in poor condition down-regulate MUP excretion^20–23^ (see more below). Third, body mass had no influence on male urinary protein excretion, indicating that MUP excretion is not an index of size. Neither body mass nor age correlated with social status, unlike some rodent studies^48,68^, though such correlations may be a consequence of social rank influencing access to food, rather than vice versa^69,70^. Our results align with other studies that find no relationship between body mass and social status or urinary protein excretion^44,45^, though the correlation between size and social rank may occur in *Mus musculus domesticus*, and not *Mus musculus musculus*^71^. Fourth, MUP upregulation by territorial males was relatively stable over the 12 weeks of living in seminatural conditions (Supplementary Fig. S12), and it was reversible, as it returned to levels similar to caged-controls after removing social interactions.

Females did not regulate protein excretion according to social status, contrary to a previous suggestion^62^, though our findings confirm that protein excretion is sexually dimorphic in seminatural as well as laboratory conditions^5,14,15^. Sex differences in MUP excretion were less pronounced in the enclosure mice than controls in standard conditions during the enclosure phase (male:female was 5:1 in enclosures versus ca. 7:1 in cages; Supplementary Table S7). This result is explained by female enclosure mice showing an increase in total urinary protein excretion under seminatural conditions. A previous study observed an increase in protein excretion (PC ratio) of females housed in mixed-sex groups and subjected to brief social interactions with unfamiliar individuals^72^. Thus, our results confirm sex differences in urinary MUP excretion, though they were not as large in laboratory conditions here or in previous studies (10:1 in *Mus m*. *musculus*^15^; three- to four-fold differences in *Mus m*. *domesticus*^5^). Future studies on chemosensory sex recognition^73^ should therefore consider the possibility that sex differences might be inflated in the laboratory.

The mechanisms controlling the regulation of MUP excretion and their adaptive functions are unclear. Urinary MUPs are transcribed in the liver and expression is under complex endocrine control, involving several hormones and pulsatile secretion^16,17,74^. Therefore, behaviors associated with social dominance, such as winning fights and scent-marking, may upregulate MUP expression by triggering pulsatile hormonal secretions. Unfortunately, previous studies have focused on physiological and behavioral changes following social defeat rather than winning^57,75^, whereas our findings cannot be explained by subordinates losing fights^26^, having poorer health, nutrition, or condition^21–23^. Regardless of the physiological mechanism, there are several possible adaptive benefits to upregulating the amount or type of MUPs excreted for dominant males, which include (1) enhancing female olfactory attraction; (2) prolonging the release of VOCs from a male’s scent marks^7,76^, which may extend their apparent freshness and enhance pheromonal effects on female physiology^3^; (3) increasing the aversiveness of a male’s territory to rival males^32^; (4) regulating energy expenditure and metabolism^77^; and (5) facilitating toxic waste excretion^78–80^. These hypotheses are not mutually exclusive, and we investigated the first hypothesis.

Our olfactory experiments showed that estrous female mice were more attracted to the scent of dominant than subordinate males, which is consistent with previous studies using dyadic agonistic assays as proxies for social status^33,34^. Female olfactory preferences were expressed only during estrus, suggesting a mating function. The lack of preference by diestrous females can be explained by cyclical silencing of vomeronasal sensory neurons, which makes female mice “blind” to certain male pheromones during diestrus and restores discrimination during estrus^81^. Female preferences may have been mediated by MUPs, VOCs, or both (as MUPs were correlated with VOC intensity, and females were able to contact the urinary stimuli before entering the arms of the Y-maze). We presented females with samples of male urine *collected from the same individual* that differed in protein concentration – at levels comparable to the differences between dominant and subordinate males – and surprisingly, females showed no preference for high protein concentration. This result means that differences in the amount of urinary protein concentration *per se* are not sufficient to explain female olfactory preferences for dominant males (even though females can discriminate larger differences in MUP quantities, such as for sex recognition^82^). Therefore, we conducted proteomic and GC-MS analyses to test for differential regulation of particular MUP proteoforms and changes in known pheromones.

We applied high-resolution mass spectrometry^25^ to determine whether urinary protein regulation was consistent for all MUP proteoforms, or whether only specific MUPs were regulated depending on social status. We did not observe any difference between dominant and subordinate mice in relative spectra intensities of seven identifiable proteoforms (MUP1-MUP5, MUP17, and MUP20). However, differences between dominant and subordinate males became evident when we compared within-individual fold changes of MUP proteoform production. Dominant males significantly increased the excretion of four MUP proteoforms (MUP2, MUP5, MUP17, and MUP20) in social conditions, whereas subordinate mice showed no significant fold changes. These results indicate that dominant male house mice show differential upregulation of specific MUP proteoforms.

Upregulating particular MUP proteoforms may function as a mechanism for dominant males to elicit specific information or responses from conspecifics. Adding recombinant MUP3 to male urine, for example, promotes aggression from male conspecifics^83^. Furthermore, MUP20 is predominately expressed in male urine (though it is also found in utero-vaginal secretions^84^), it is a female attractant^66^, and it mediates spatial learning^85^ by stimulating neurogenesis^86^. MUP20 also stimulates male-male aggression^83^ and maternal aggression from lactating females^87^. Studies are needed to determine whether honest signaling is enforced by punishment of dishonest signalers (‘social control’ or punishment hypothesis^88^). Our findings support previous studies of *Mus musculus domesticus* showing increased MUP20 production in relation to social status after a three-day^44^ and a 17-day enclosure experience^45^ in highly male-biased populations. Taken together, these studies suggest that upregulation of specific MUP proteoforms provides a reliable indicator of dominant male social status, which may deter challenges from subordinate rivals, as well as attract potential mates.

MUPs control the binding of volatile male pheromones, but there are surprisingly few studies on the regulation of MUPs on volatiles – and none in natural or seminatural conditions. Our previous analyses verified the presence of male pheromones in our enclosure mice^80^, and we expected that elevating MUP excretion would increase the intensities of volatile pheromones and other ligands in urine. Indeed, we found a strong, positive correlation between urinary protein concentration and intensity of VOCs, and denaturing the proteins increased the overall ion intensities of all VOCs detected and analyzed (peak heights in the TIC, Fig. 4). This result supports the hypothesis that increasing MUP concentration increases the amount of pheromones and other VOCs in the urine. It suggests that by increasing MUP excretion, dominant males should be able to prolong the attachment of their pheromones to substrate and subsequently extend the release of volatiles over time. We expected that we might detect differences in the amount of unbound VOCs, and particularly, pheromones previously found to correlate with male rank^39,44,46^. However, male social status did not affect urinary VOC profiles, except for hydroxy-6-methyl-3-heptanone (HMH), which showed the highest relative intensity in dominant males. HMH is an androgen-dependent volatile compound previously found to accelerate female puberty in some laboratory strains^42,64,65^. HMH intensities are higher in males than in females, and females prefer HMH when mixed with DHB and SBT, but not HMH alone^42^. Thus, elevated HMH levels in dominant males might function to attract adult females and accelerate sexual maturity in peripubertal females. Elevated HMH levels might explain female preferences for dominant males’ scent in the olfactory discrimination assay. We compared the intensities of other male pheromones, including 3,4-dehydro-exo-brevicomin (DHB), 2-sec-butyl-4,5-dihydrothiazole (SBT) and β-farnesene, and found no significant differences between dominant and subordinate males. The intensity of SBT was higher in caged controls than enclosure males, supporting results that compared isolated versus group-living mice in laboratory conditions^89^. These findings raise additional caveats about studying chemosignals in standard laboratory conditions. We found no evidence for female-specific VOCs (denaturation of female urine released mainly flavor and fragrance compounds which were potentially derived from food), and VOC profiles of dominant and subordinate females were almost indistinguishable. It is surprising that we did not detect more compounds that differ between dominant and subordinate males, but MUPs show extremely strong binding affinity for male pheromones, and so some ligands remain bound, even after extensive purification^90^. Thus, it is possible that GC-MS will fail to detect actual differences in the amounts of these bound ligands.

## Conclusions

Male mice increased the excretion of major urinary proteins (MUPs) once they acquired a territory and became socially dominant, which may function to attract females. Estrous females were more attracted to the scent of dominant than subordinate males, however, they were not attracted to the scent of urine with elevated MUP concentration when male social status was controlled. Dominant males also differentially upregulated specific MUPs, including (MUP20) and a volatile pheromone (HMH), a MUP-ligand that may influence female behavior and reproduction. Future studies are needed to examine the proximate mechanisms through which dominant males regulate these pheromones and how they influence mating success. Since social status of male house mice is influenced by phenotypic and genetic quality (e.g., inbreeding)^49^, our findings suggest that MUP excretion provides a reliable indicator of genetic quality to potential mates.

## Methods

### Trapping, housing and breeding animals

Experimental animals were F1 offspring of wild-caught house mice (*Mus musculus musculus*) trapped at seven locations within a 300 m radius in Vienna, Austria (48°13′14″N; 16°17′00″E) and crossed between trapping sites to avoid inbreeding. F1 mice were weaned at 21 ± 1 d, separated from siblings at the age of 35 ± 1 d and individually housed in standard mouse cages (type IIL, 36.5 × 20.5 × 14 cm, Tecniplast, Germany) containing wooden bedding (ABEDD, Austria), a cardboard roll, cotton nestlets^©^ (ABEDD, Austria), and a plastic nest box (Tecniplast, Germany). Food (Altromin rodent diet 1324) and water were provided *ad libitum* and temperature was maintained at 22 ± 2 °C. Mice were kept on a 12:12 h light:dark cycle with red lights on at 1500. The authors use these descriptions of housing, diet, and light:dark cycle to define standard housing conditions. At weaning, all animals received an ear-punch for individual identification and to obtain tissue for DNA extraction. The Ethical and Animal Welfare Commission at the University of Veterinary Medicine Vienna approved the experimental protocols (Permit No. 02/08/97/2013). We confirm that all experiments and animal handling were performed according to the ethical standards and guidelines outlined by the Ethical and Animal Welfare Commission.

### Housing conditions: seminatural populations and caged controls

F1 mice were assigned to either *enclosure treatment* (N = 64, 1:1 sex ratio) or *caged control* group (N = 64, 1:1 sex ratio). Mice in the enclosure group were assigned to one of eight replicate populations, and each population had eight mice (1:1 sex ratio, and genetically unrelated, except each mouse had one littermate of the opposite sex in the population). Males within populations were matched for body mass (range = 0.6 g). To test whether initial urinary protein excretion predicted males’ subsequent social status, we also assigned males depending on their relative urinary protein production (sampled 4 weeks before release into enclosures; see below), and each population contained one high, two intermediate, and one low urinary protein producer (see Supplementary Table S1). F1 animals were sexually mature at the time of initial urine collection (age in days of males = 183 ± 22 s.d.; females = 185 ± 20 s.d.). Seminatural enclosures (8 enclosures; 3.4 × 4 m each) were subdivided into six equally-sized compartments by fencing (wire mesh, 40 cm high), which the mice could scale but tended to use as territorial boundaries. Each enclosure contained wooden bedding (ABEDD, Austria), plastic nest boxes, a water station, wood wool, and paper towels as nesting material. Food (Altromin rodent diet 1324) and water were provided *ad libitum* and temperature was maintained at 22 ± 2 °C. Mice were kept on a 12:12 h light:dark cycle with white light off (and red lights on) at 1500. The caged controls were litter-mates of mice in the enclosure group, and were housed under standard colony conditions during the experiment.

### Behavioral observations

To assess social status, behavioral observations were recorded three to five days per week for 30 min/d between 1500 and 1700 (peak activity) for 12 weeks (161.5 h total observation time, 20.2 h mean time per population). Before release into the enclosures, males received unique fur cuts, allowing easy identification under red light; females were identified by unique ear punches. Observers recorded interactions (aggressive, submissive, and investigatory actions), the location of the interaction, and the mice involved. A dominance index for each mouse was calculated as (number of aggressive + investigatory interactions)/(total number of interactions involving the individual)^49^. Mice obtaining a dominance index ≥80% within an enclosure compartment were considered to be dominant; otherwise they were considered subordinate. Adult survival was monitored daily and offspring born in the enclosures were removed upon discovery.

### Urine sampling

For monitoring urinary protein and pheromone production, we collected six urine samples from each mouse over 20 weeks at four week intervals (Supplementary Fig. S1): two times before release into the enclosures (‘before enclosure’), three times during housing in the enclosures (‘during enclosure’), and once after termination of the enclosure treatment (‘after enclosure’). Control mice were sampled on the same schedule. We collected urine using metabolic cages (Techniplast, Germany) to minimize handling stress and fecal contamination. Collections were conducted under red light at the beginning of the dark cycle. To stimulate urination, female urine (5 µl of urine pooled from 4 females) was placed onto filter paper (4 cm^2^) and then hung inside the metabolic cage. Upon excreting >70 µL of urine, mice were either returned to their home cage (cage controls) or released back into the enclosure (during the enclosure phase). Only 2% (17/700) of urine collections provided an insufficient volume during the 60-min sampling period, in which case mice were returned to their cage or enclosure without sampling. Urine and feces were transferred to separate Eppendorf tubes or glass vials (40 µL for GC-MS analyses), immediately frozen, and stored at −80 °C. As part of our experimental design, storage times differed depending on collection date, but handling was the same for each sample to avoid possible biases. Previous studies used a subset of these samples to compare the effects of housing (standard versus seminatural) on urinary volatiles^80^ and proteins^25^.

### Olfactory discrimination assays

We investigated female olfactory preferences using a two-choice Y-maze assay. The female ‘sniffers’ (subjects) were unrelated, wild-derived mice (F4, age 172 ± 10 d). Subjects were housed with a sister in our animal facility, and they were unfamiliar to urine stimulus donors. Estrous cycling was stimulated by introducing male bedding into the home cage of test mice and daily vaginal smears were performed to determine estrus (signified by >90% cornified epithelial cells^91^). The Y-maze apparatus consisted of clear PVC tubes (5 cm Ø) and a PVC connector (Supplementary Fig. S2). The length of the primary branch was 20 cm and the secondary branches were 15 cm long. Each secondary branch opened into a circular chamber (PVC, 16 cm Ø; 8 cm height) and a clear plexiglass plane was placed under and on top of the chamber to prevent escape. The chamber allowed observation of sniffing behavior and interaction with the stimulus at the end of the tube, in an open area. A test mouse entered the Y-maze from a start cage (type II, 26.8 × 21.5 × 14 cm, Tecniplast, Germany) with clean bedding and connected to the primary branch with a short piece of clear PVC tubing (4 cm Ø; 10 cm long). The start cage was equipped with a remote-controlled metal door. The Y-maze was divided into 5 zones: a primary branch zone, left and right secondary branch zone and left and right chamber zone. A mouse was considered to be in a zone when its body (nose-tail base) was observed in that zone. All trials were video recorded from an aerial perspective (110 cm above center of Y-maze; D-Link 3710DCS IP-camera with frame rate of 25FPS). Video files were analyzed using Ethovision and The Observer software (Noldus Information Technologies, The Netherlands). We calculated the difference of left and right zones for the following variables: (1) latency to visit (s); (2) total duration in zone (s); (3) frequency of visits; (4) latency to sniff the chamber zone stimulus (s); (5) sniff duration (s); and (6) sniff frequency.

Ten minutes prior to each trial a subject was removed from its home cage, placed in the start cage, and left to acclimate in an adjacent room. Our design differed from conventional Y-mazes because in addition to placing odor stimuli at the ends of the secondary branches, we also placed urine in the decision zone at the fork, where the primary and secondary branches meet. We transferred 3 µl of urine to the floor of the decision zone, 2.5 cm before the secondary branches, which allowed subjects to sniff and contact both stimuli before deciding to enter one of the two branches (Supplementary Fig. S2). An additional 3 µL aliquot was placed on the floor of the chamber 15 cm across from the secondary branch, allowing us to observe direct sniffing of the stimulus in a relatively open space. The urinary stimuli in the decision zone and chambers were spread over a 1 cm^2^ area using a pipette tip. The start cage was connected to the Y-maze, experimenters left the room and the metal door was remotely opened. A 5-min trial began once the subject entered the primary branch zone. All mice voluntarily entered the maze during test trials and explored all branches, but not always the chamber zones. At the end of each trail, the subject was returned to its home cage. Y-mazes were washed with odorless soap and hot water and allowed to dry for 24 h between trials. The plexiglass planes and metal entry door were cleaned with 70% ethanol and allowed to dry between trials.

We compared the following stimuli in our olfactory discrimination experiments: (1) adult male urine versus water (‘positive control’ to confirm that females in our assay are attracted to the scent of male urine^61^; N = 15 subjects in estrus; pooled male urine); (2) adult male versus adult female urine (‘sex discrimination’; N = 10 subjects in estrus; pooled urine of same-sex individuals); (3) dominant males versus subordinates (‘social status discrimination’; N = 10 subjects tested during the estrous and diestrous phases; male urine from enclosure treatment); and (4) high versus low urinary protein concentration (‘concentration discrimination’; N = 10 subjects in estrus; urine from the same male donor (N = 10) collected on two different days; 3:1 high:low PC ratio, see below).

### Proteomic analyses

#### Total and adjusted urinary protein excretion

Total urinary protein excretion (mg ml^−1^) was measured in triplicates using a standard Bradford assay^92^ on a 96-well microplate. Triplet values occurred within ±10% range, otherwise the analysis was repeated. We used total urinary protein excretion and adjusted with creatinine concentration to calculate urinary protein excretion (protein:creatinine ratio or PC ratio), as this value is expected to account for renal activity and urine dilution (InVitro: Labor für Veterinärmedizinische Diagnostik & Hygiene GmbH, Vienna). We confirmed that MUPs comprise most (85 ± 7%) of the total protein in the urine of these mice in a preliminary study^25^. We provide results for PC ratio, total urinary protein (unadjusted values), and creatinine.

#### Protein identification and quantification using SWATH

Urine samples of 12 dominant and 5 subordinate males were obtained during the enclosure phase and pooled for each individual for protein analysis. As previously described, protein identification and quantification was conducted using a label-free proteomics technique known as SWATH^25^. Relative quantification was conducted using two different databases containing (1) all reviewed proteins from Swissprot, and (2) 9 reviewed MUP proteins from Uniprot. In total, 42 different MUP sequences were found in Uniprot, though many of these were not distinguishable using a bottom up approach due to signal peptide cleavage at protein maturation. Thus, we employed a conservative approach by including only reviewed MUPs which have been identified independently by at least two different working groups.

### GC-MS analysis of volatile molecules

Urine samples of 25 males (17 enclosure mice (12 dominant, 5 subordinate) and 8 caged controls) and 15 females (10 enclosure mice (5 dominant, 5 subordinate) and 5 caged controls) were obtained during the enclosure phase and pooled for each individual. As previously reported, 15 µL of intact or denatured urine were analyzed using gas chromatography coupled with mass spectrometry (GC-MS)^80^. Compounds were identified using mass spectral library comparison (NIST08) combined with manual interpretation. Exact peak alignment was difficult during the first 5 min of each run, which resulted in a wide peak detection window and increased chances of aligning separate peaks into one. Thus, the dataset suffered from poor peak alignment and difficulties with identifying compounds due to co-elution. After leaving out the first 5 min of each run, we were able to use a significantly smaller peak detection window, which improved dataset quality and enabled reliable peak identification. However, information on the first 5 min of each run (and differentiating compounds therein) is missing from this dataset.

### Statistical analyses

Statistical analyses were performed using the R statistical package (R Development Core Team 2014 version 3.4.4^93^) and the assumptions of the methods used were first verified.

#### Urinary protein excretion

We used linear mixed effects models (LME, *lme* function, package *nlme*^94^) to examine the relationship between urinary protein excretion (PC ratio, total protein excretion, and creatinine excretion) and social status, we compared three ‘social status’ groups: dominants, subordinates, and caged controls in standard housing conditions. PC ratio and creatinine values were natural log transformed to achieve a normal distribution. Social status, enclosure phase (before, during, and after enclosure), and the interaction of social status * enclosure phase were used as fixed factors; age and body mass were covariates in each model. The random factor was composed of individuals nested in population. We used the *varIdent* function to account for heteroscedasticity in the social status factor and fit the LME model using the maximum likelihood method. The *anova*.*lme* function produced F and p values for testing significance of model variables. Pairwise comparison *post hoc* tests were performed for the interaction social status * enclosure phase in each model using the *glht* function with Tukey correction for multiple testing (package *multcomp*^95^).

Sex differences were examined using generalized linear mixed models (GLMMs, *glmer* function, package *lme4*^96^) with sex, housing, and the sex * housing interaction as fixed effects on the dependent variables of PC ratio, total urinary protein, and creatinine excretion measured during the enclosure phase (three separate models). Mouse ID and population were included as random effects. An inverse link function was used to account for the inverse Gaussian distribution of the urinary protein variables. Wald chi-square tests^97^ were used to determine significance of the model effects (function *anova*, package *stats*^93^). Associations between body mass parameters (initial mass, mean mass, and change in mass) and social status of enclosure males were examined using Mann-Whitney U tests. Due to pregnancy effects, females were excluded from mean mass and change in mass comparisons.

#### Olfactory discrimination assays

When normality of the variables was not rejected (Shapiro-Wilks test), One-Sample T-tests (μ = 0) were used for the positive control, sex, and concentration discrimination experiments. Wilcoxon Signed Rank Tests were used for non-normal data to examine a deviation from a zero median. Fisher Exact test (1:1 null expectation) was used to determine a significant bias in initial preference. To account for the paired design for the social status discrimination experiment, McNemar test was used to compare initial preferences, and linear mixed-effects (LME) models (*lmer* function, package *lme4*^96^) were used to measure frequency, duration, and latency for each zone (if necessary, variables were log-transformed (sign preserved) to normalize model residuals). Estrous state of the subjects was included as a fixed factor; subject ID and male stimulus donors’ ID were included as random factors. Models were fitted by residual maximum likelihood and the *anova* function produced F and p values. Benjamini-Hochberg method (*p*.*adjust* function) was used to control multiple hypothesis tests for false discovery rate.

#### Multivariate analyses of urinary proteins and MUP-bound volatiles

Analyses were carried out on the relative intensity and protein amount of 7 MUPs and 115 non-MUP proteins detected using SWATH. Relative intensity for each compound was calculated by dividing the peak intensity of each compound by the total chromatogram intensity. Protein amount of each compound was calculated by multiplying the relative intensity of each compound by the total amount of protein in the sample (determined by Bradford assay). To investigate the effects of social experience on urinary protein excretion, we calculated the difference in protein amount and relative intensity from X_during_ − X_before_. Fold change in protein amount and relative intensity was calculated from (X_during_ − X_before_)/X_before_ and compared between social status groups as described below. Additionally, fold change in protein amount and relative intensity of MUPs was compared to null expectations using One-Sample T-tests with Benjamini-Hochberg correction. To investigate changes in concentration of individual MUPs from introducing singly housed males into a social setting (enclosures), we ran a multivariate general linear model with individual MUP protein concentrations (obtained from SWATH quantification, see above) as continuous variables and social status (dominant, subordinate), enclosure phase (before vs during), and the interaction of social status * enclosure phase as factors. Age and body mass were used as covariates in the model. The random factor was composed of individuals nested in population. To investigate whether concentration of individual MUPs before introduction into enclosures were predictive of male social status, we used Mann-Whitney U tests with Bonferroni correction.

Analyses were also carried out on the relative intensity of MUP-bound volatiles obtained by GC-MS. We ran an exploratory data analysis (untargeted analysis of all ions of the peaks detected in each sample) on a dataset containing peak ion intensities (N = 962) after leaving out the first 5 min of each run. Second, we took a targeted approach by analyzing manually collected peak intensities of 34 known urinary volatiles for each sample (see Supplementary Table S2 for targeted compound names and potential functions).

#### Patterns of chemical similarity

Chemical profile similarity was visualized using nonmetric multidimensional scaling (NMDS) based on pairwise Bray–Curtis similarity values calculated from the log(x + 1)- transformed data of protein amount or relative intensity. NMDS allows visualization of high-dimensional chemical similarity by placing each individual in a 2D scatter plot such that points closer together represent individuals with relatively high chemical similarity. The grouping structure of individuals according to their social status was analyzed using nonparametric ANOSIM, a permutation test to evaluate differences between two or more groups without assumptions about data distribution or homoscedasticity. A significant test result indicates that the chemical similarity within the groups is significantly greater than the chemical similarity between the groups. These analyses were implemented using the *vegan* package^98^.

#### Identification of differentiating compounds

To identify compounds that differentiate between social status groups, we used Sparse Partial Least Squares Discriminant Analysis (SPLS-DA) with subsequent univariate test. SPLS-DA is a classification method appropriate for data with a high number of potentially correlated variables and needs no preliminary variable filtering^99^. Intrinsic variable selection was performed to filter out relevant variables, and the data was projected onto uncorrelated latent variables. These linear combinations of the relevant variables were then used in linear discriminant analysis to predict class membership. The package *spls*^100^ provides an implementation of SPLS-DA, namely the function *splsda*. The optimal model parameters were searched for with the function *cv*.*splsda* and the number of latent variables (with possible values 1, 2, …, 10) and the sparsity parameter (with possible values 0.1, 0.2, …, 0.9) were selected with 5-fold cross validation.

We ran 16 classification models to identify urinary proteins that differentiated the social status groups based on the following variables: (1) relative intensity; (2) protein amount; (3) difference (during - before enclosure) in relative intensity; (4) difference in protein amount; (5) fold change in relative intensity; and (6) fold change in protein amount. Additionally, for intact and denatured urine samples, 12 classification models were estimated to obtain volatile urinary compounds that differentiated between the social status groups and housing conditions (see Supplementary Table S3 for summary of classification models and their misclassification rates). Univariate tests were applied to all variables selected by SPLS-DA and tested with Benjamini-Hochberg correction for multiple testing.

## Supplementary information

Supplementary Information

## Data Availability

The full datasets will be made available upon publication (protein sequences will be submitted to the PRIDE repository).
