# Author Correction: Regulation of volatile and non-volatile pheromone attractants depends upon male social status

**DOI:** 10.1038/s41598-019-41666-4

**Published:** 2019-04-11

**Authors:** M. Thoß, K. C. Luzynski, V. M. Enk, E. Razzazi-Fazeli, J. Kwak, I. Ortner, D. J. Penn

**Affiliations:** 1 Department of Integrative Biology and Evolution, Konrad Lorenz Institute of Ethology, University of Veterinary Medicine, Vienna, Austria; 2 Proteomics Unit, VetCORE Facility for Research, University of Veterinary Medicine, Vienna, Austria; 3 Department of Integrative Biology and Evolution, Research Institute of Wildlife Ecology, University of Veterinary Medicine, Vienna, Austria; 4 Institute of Statistics and Mathematical Methods in Economics, TU Wien, Vienna, Austria; 5 Present Address: International Flavors & Fragrances Inc., Union Beach, New Jersey USA; 6 Present Address: Department of Mathematics, Statistics Section, KU Leuven, Leuven, Belgium

Correction to: *Scientific Reports* 10.1038/s41598-018-36887-y, published online 24 January 2019

The Acknowledgements section in this Article is incomplete.

“We are grateful to T. Klaus, L. Hoelzl, D. Gliga, D. Krebber, C. Schüssele, and D. Reitschmidt for excellent support in conducting experiments. We thank A. Sasse and U. Madlsperger for animal care, and I Miller, K. Nöbauer, and K. Hummel for technical support. We thank E. Straßer and M. Hämmerle for assistance with GC-MS analyses, and T. Ruf for statistical advice. S. Zala, P. Stopka, and an anonymous reviewer provided helpful comments on the manuscript.”

should read:

“We are grateful to T. Klaus, L. Hoelzl, D. Gliga, D. Krebber, C. Schüssele, and D. Reitschmidt for excellent support in conducting experiments. We thank A. Sasse and U. Madlsperger for animal care, and I Miller, K. Nöbauer, and K. Hummel for technical support. We thank E. Straßer and M. Hämmerle for assistance with GC-MS analyses, and T. Ruf for statistical advice. S. Zala, P. Stopka, and an anonymous reviewer provided helpful comments on the manuscript. This work was funded by the Austrian Science Fund (FWF): P24711-B21. The funder had no role in study design, data collection and analysis, decision to publish, or preparation of the manuscript.”

