# Supplementary material for: Regulation of volatile and non-volatile pheromone attractants depends upon male social status
Source: Sci Rep. 2019 Jan 24;9:489. doi: 10.1038/s41598-018-36887-y (PMC6346026; doi:10.1038/s41598-018-36887-y)
Supplement: Supplementary file 1 — Supplementary Information [file 41598_2018_36887_MOESM1_ESM.pdf]

Regulation of volatile and non-volatile pheromone attractants depends upon male social status

M. Thoß[1¶], K.C. Luzynski[1¶], V.M. Enk[2], E. Razzazi-Fazeli[2], J. Kwak[3#a], I. Ortner[4#b], and D. J. Penn[1]*

[1] Department of Integrative Biology and Evolution, Konrad Lorenz Institute of Ethology, University of Veterinary Medicine, Vienna, Austria

[2] Proteomics Unit, VetCORE Facility for Research, University of Veterinary Medicine, Vienna, Austria

[3] Department of Integrative Biology and Evolution, Research Institute of Wildlife Ecology, University of Veterinary Medicine Vienna, Vienna, Austria

[4] Institute of Statistics and Mathematical Methods in Economics, TU Wien, Vienna, Austria

[#a]Current Address: International Flavors & Fragrances Inc., Union Beach, New Jersey, USA

[#b]Current Address: Department of Mathematics, Statistics Section, KU Leuven, Belgium

* Corresponding author

dustin.penn@vetmeduni.ac.at

[¶]These authors contributed equally to this work.

Regulation of volatile and non-volatile pheromone attractants depends upon male social status

Supplementary Tables S1 to S11

Table S1. Study mice information at the time of first urine collection as well as social status.

pop	sex	ID	Age at initial urine collection (days)	Mass at initial urine collection (grams)	PC ratio of initial urine collection	PC rank of initial urine collection	Dominance Index	Social status based on Dominance Index	Aggressive Interactions	Investigatory Interactions	Submissive Interactions
A	m	K17.4	198	27.4	13.36	High	0.85	DOM	127	17	26
A	m	K21.5	159	27.6	2.60	Low	0.23	SUB	14	17	106
A	m	K24.6	193	27.4	4.17	Intermediate	0.94	DOM	140	26	11
A	m	K29.5	193	27.1	5.61	Intermediate	0.21	SUB	6	9	56
A	f	K17.1	198	18.9	3.29		0.74	SUB	4	13	6
A	f	K21.1	159	24.6	0.91		0.56	SUB	10	8	14
A	f	K24.2	193	17.7	2.34		0.08	SUB	1	8	101
A	f	K29.1	193	26.6	3.54		0.92	DOM	95	25	10
B	m	K19.5	196	25.8	12.38	Intermediate	0.32	SUB	15	3	39
B	m	K28.4	195	25.1	8.42	Intermediate	0.27	SUB	11	7	49
B	m	K29.7	193	25.6	4.32	Low	0.91	DOM	140	17	16
B	m	K30.9	193	25.8	23.22	High	0.80	DOM	70	15	21
B	f	K19.1	196	22	3.31		0.23	SUB	4	11	50
B	f	K28.2	195	21.2	7.60		0.31	SUB	3	2	11
B	f	K29.2	193	26.4	1.86		0.94	DOM	14	16	2
B	f	K30.1	193	19.4	2.32		0.48	SUB	6	9	16
C	m	K16.5	192	23.5	7.27	Intermediate	0.39	SUB	5	10	23
C	m	K22.5	199	23.8	5.54	Low	0.96	DOM	163	98	12
C	m	K4.8	184	23.5	25.10	High	0.94	DOM	176	107	17
C	m	K9.2	202	23.8	11.73	Intermediate	0.29	SUB	3	10	32
C	f	K16.2	192	20.6	1.89		0.90	DOM	136	75	23
C	f	K22.2	199	22.7	1.53		0.90	DOM	93	61	18
C	f	K4.3	184	20.4	2.44		0.23	SUB	5	42	154
C	f	K9.1	202	23.5	3.76		0.36	SUB	16	49	117
D	m	K12.8	145	23.1	6.51	Intermediate	0.67	SUB	0	6	3
D	m	K2.5	200	23.4	3.34	Low	0.00	SUB	0	0	0
D	m	K24.5	193	23.2	11.70	High	0.38	SUB	4	5	15
D	m	K26.6	152	23.4	4.68	Intermediate	0.98	DOM	62	214	5
D	f	K12.2	145	17.3	1.83		0.61	SUB	24	125	97
D	f	K2.1	200	19.2	0.70		0.22	SUB	2	21	82
D	f	K24.1	193	21.8	2.53		0.51	SUB	6	101	102
D	f	K26.3	152	19.7	4.76		0.98	DOM	219	86	5
E	m	K10.6	204	22.9	9.88	Intermediate	1.00	DOM	79	121	0
E	m	K13.6	200	22.3	12.69	High	0.32	SUB	2	16	38
E	m	K28.5	195	22.6	5.23	Low	0.06	SUB	0	1	17
E	m	K4.2	209	22.9	9.04	Intermediate	0.00	SUB	0	0	0
E	f	K10.1	204	16.2	0.40		0.75	SUB	39	37	25
E	f	K13.3	200	17.3	1.47		0.18	SUB	2	29	143
E	f	K28.1	195	20.1	19.74		0.98	DOM	117	71	4
E	f	K4.1	209	19.1	3.39		0.87	DOM	3	106	17
F	m	K11.5	204	22.2	6.41	Intermediate	0.00	SUB	0	0	1
F	m	K26.4	152	22	28.99	High	0.12	SUB	0	3	23
F	m	K27.4	193	22.1	8.90	Intermediate	0.14	SUB	2	2	25
F	m	K30.8	193	22.3	4.63	Low	0.98	DOM	124	58	3
F	f	K11.2	204	20	4.74		0.69	SUB	4	5	4
F	f	K26.1	152	19.6	0.74		0.77	SUB	14	6	6
F	f	K27.1	193	20.1	2.06		0.11	SUB	3	11	114
F	f	K30.2	193	21.3	3.12		0.94	DOM	45	20	4
G	m	K18.9	198	21.3	10.10	Intermediate	0.40	SUB	6	34	60
G	m	K21.6	159	21.6	1.54	Low	0.43	SUB	7	30	49
G	m	K3.3	208	21.9	5.70	Intermediate	0.98	DOM	122	30	3
G	m	K8.2	204	21.5	12.19	High	0.82	DOM	64	58	26
G	f	K18.1	198	20.4	2.84		0.30	SUB	0	13	31
G	f	K21.2	159	22.4	0.69		0.81	DOM	10	32	10
G	f	K3.1	208	20.7	1.34		0.62	SUB	6	15	13
G	f	K8.1	204	23.3	0.79		0.74	SUB	2	38	14
H	m	K1.9	155	19.4	3.38	Low	0.40	SUB	1	3	6
H	m	K13.5	200	19.5	3.82	Intermediate	0.83	DOM	21	22	9
H	m	K22.4	199	19.8	8.34	Intermediate	0.97	DOM	41	30	2
H	m	K27.6	193	19.5	9.80	High	0.57	SUB	0	4	3
H	f	K1.2	155	20.2	0.70		0.75	SUB	0	6	2
H	f	K13.2	200	16.8	1.85		0.27	SUB	3	10	36
H	f	K22.1	199	16.6	0.83		0.88	DOM	47	17	9
H	f	K27.3	193	21.6	0.99		0.40	SUB	2	20	33
lab	m	K10.10	204	23.1	4.24						
lab	m	K10.9	145	23.7	3.49						
lab	m	K11.4	122	22.5	9.42						
lab	m	K12.9	166	21.1	6.96						
lab	m	K14.1	185	21.8	14.24						
lab	m	K14.2	158	25.8	7.16						
lab	m	K14.3	158	23.5	4.96						
lab	m	K16.3	199	22.4	5.67						
lab	m	K16.4	159	24.2	4.87						
lab	m	K17.2	159	23.2	8.02						
lab	m	K17.3	166	23	4.58						
lab	m	K18.15	103	16.9	3.32						
lab	m	K18.16	193	25.6	3.83						
lab	m	K19.7	165	27	4.76						
lab	m	K20.2	184	20.2	4.57						
lab	m	K21.12	202	21.9	3.20						

lab	m	K23.4	173	23.4	5.26						
lab	m	K24.7	174	23.2	11.54						
lab	m	K25.3	207	21.1	6.31						
lab	m	K25.5	164	24.6	7.00						
lab	m	K26.5	185	22.3	5.95						
lab	m	K27.5	192	25.9	14.63						
lab	m	K28.3	195	26.9	4.99						
lab	m	K3.8	187	18.4	7.13						
lab	m	K30.7	192	24.2	4.06						
lab	m	K4.9	195	22.2	23.48						
lab	m	K5.2	195	23.1	3.79						
lab	m	K5.3	180	25.9	13.99						
lab	m	K7.4	202	24.7	9.71						
lab	m	K7.6	193	18.7	13.06						
lab	m	K8.3	202	27.7	4.29						
lab	m	K9.3	193	24.3	5.23						
lab	f	K1.3	155	16.9	1.54						
lab	f	K10.3	204	20.5	1.62						
lab	f	K11.1	200	16.8	2.33						
lab	f	K12.1	192	18.8	1.76						
lab	f	K13.1	166	16.2	1.64						
lab	f	K14.7	158	18.6	2.50						
lab	f	K16.1	159	18.1	2.24						
lab	f	K17.8	166	21.4	1.63						
lab	f	K17.9	152	19.8	1.66						
lab	f	K20.1	165	20	1.51						
lab	f	K20.6	207	19.5	1.90						
lab	f	K20.7	180	19.7	1.47						
lab	f	K20.8	180	21.3	1.75						
lab	f	K21.3	178	20.6	2.01						
lab	f	K22.3	178	17.2	2.02						
lab	f	K23.8	131	15.1	1.46						
lab	f	K23.9	164	21.3	1.43						
lab	f	K25.8	193	19.3	1.46						
lab	f	K25.9	174	18.3	1.75						
lab	f	K26.2	204	19.6	1.46						
lab	f	K26.7	198	19.3	2.52						
lab	f	K27.2	196	17.3	1.55						
lab	f	K27.7	201	19.7	1.69						
lab	f	K28.8	202	20.9	1.85						
lab	f	K4.4	152	18.8	2.40						
lab	f	K5.1	145	20	1.74						
lab	f	K5.5	201	29.7	1.65						
lab	f	K5.6	198	21.2	2.93						
lab	f	K7.2	204	20.1	1.79						
lab	f	K8.7	207	21.2	2.97						
lab	f	K8.8	201	23.1	1.98						
lab	f	K9.5	184	21.9	9.84						

Table S2. Mouse urinary compounds selected for targeted approach GCMS analysis

**Substance name**	**Function**	**Reference**	**Previously found in mouse urine by**
Trimethylamine	male-only compound	Liberles & Buck 2006	Röck et al 2006
Isovaleraldehyde	flavor agent		Röck et al 2006
2-Pentanone	flavor agent		Röck et al 2006
Isopentylamine	food derivative		
3-Penten-2-one	NA		Röck et al 2006
6-hydroxy-6-methyl-3-heptanone	male pheromone	Novotny et al 1999a,b	Röck et al 2006
6-Methyl-3,5-heptadien-2-one	flavor agent		
1-Methylthio-2-propanone	flavor agent		
3-Methylcyclopentanone	NA		Schwende et al 1986, Novotny et al 1999a,b, Röck et al 2006
5-Methyl-2-hexanone	food derivative		
2-Heptanone	male pheromone	Novotny et al 1987	Schwende et al 1986, Röck et al 2006
2-Heptanone	stress signal in rats	Gutierrez-Garcia et al 2007	
4-Hepten-2-one	food derivative		Schwende et al 1986
2,5-Dimethylpyrazine	flavor agent		
(Z)-2-penten-1-yl acetate	flavor agent		Schwende et al 1986
Dimethyl sulfone	food derivative		
2-Acetyl-1-pyrroline	flavor agent		
3-Hepten-2-one	flavor agent		Röck et al 2006
4-Methyl-6-hepten-3-one	NA		
2-Isopropyl-4,5-dihydrothiazole	structurally very similar to male pheromone		Liebich et al 1977, Schwende et al 1986
3-Octen-2-one (T)	flavor agent		
2-Hexen-4-olide (T)	flavor agent		
Dihydroisophorone (T)	flavor agent		
3,4-Dehydro-exo-brevicomin (DHB)	male pheromone	Novotny et al 1999b	Schwende et al 1986, Röck et al 2006
2-sec-Butyl-4,5-dihydrothiazole	male pheromone	Novotny et al 1999b	Liebich et al 1977, Schwende et al 1986, Röck et al 2006
5,9-dimethyldeca-5,8-dien-2-one	flavor agent		
(Z)-5-Tetradecen-1-ol	saw fly pheromone	Bartelt et al 1983	
Triacetin	flavor agent		
2-Butyl-2-octenal	ant alarm pheromone	Rossi et al 1992	
Farnesene	male pheromone	Novotny et al 1999b	Röck et al 2006
4-sec-Butyl-2,6-di-tert-butylphenol	food derivative		

Table S3. Details of SPLS-DA classification models and their misclassification rates

Model	Data source	Classification model	Substances sampled	# of selected compounds	Misclassification rate	# significant compounds	Differentiating compounds
1	SWATH	Protein amount before experiment	MUPs	0	8/17	0	-
2	SWATH	Relative intensity before experiment	MUPs	3	4/17	0	see S1 Table 10A
3	SWATH	Protein amount before experiment	non-MUPs	0	5/17	0	-
4	SWATH	Relative intensity before experiment	non-MUPs	0	10/17	0	-
5	SWATH	Protein amount during experiment	MUPs	0	8/17	0	-
6	SWATH	Relative intensity during experiment	MUPs	0	2/17	0	-
7	SWATH	Protein amount during experiment	non-MUPs	0	5/17	0	-
8	SWATH	Relative intensity during experiment	non-MUPs	3	6/17	0	see S1 Table 10B
9	SWATH	Protein amount during vs before experiment	MUPs	6	0/17	6	see S1 Table 10C
10	SWATH	Relative intensity during vs before experiment	MUPs	0	5/17	0	-
11	SWATH	Protein amount during vs before experiment	non-MUPs	115	0/17	105	see S1 Table 10E
12	SWATH	Relative intensity during vs before experiment	non-MUPs	43	0/17	26	see S1 Table 10D
13	SWATH	Fold change in protein amount	MUPs	0	5/17	0	-
14	SWATH	Fold change in relative intensity	MUPs	0	8/17	0	-
15	SWATH	Fold change in protein amount	non-MUPs	0	5/17	0	-
16	SWATH	Fold change in relative intensity	non-MUPs	0	8/17	0	-
1. Exploratory data analysis							
17	GCMS	Male social status - intact urine	volatile organic compounds	9	1/17	0	-
18	GCMS	Male social status - denatured urine	volatile organic compounds	52	0/17	0	-
19	GCMS	Female social status - intact urine	volatile organic compounds	68	2/10	0	-
20	GCMS	Female social status - denatured urine	volatile organic compounds	399	1/10	0	-
2. Targeted approach (see S1 Table 2)							
21	GCMS	Male social status - intact urine	volatile organic compounds	3	1/17	1	HMH
22	GCMS	Male social status - denatured urine	volatile organic compounds	7	1/17	3	HMH
23	GCMS	Female social status - intact urine	volatile organic compounds	2	3/10	0	-
24	GCMS	Female social status - denatured urine	volatile organic compounds	13	1/10	0	-
25	GCMS	Housing conditions - Males - intact urine	volatile organic compounds	7	1/25	3	SBT, Isopentylamine, Pentanone
26	GCMS	Housing conditions - Males - denatured urine	volatile organic compounds	10	1/25	1	Octen-2-one
27	GCMS	Housing conditions - females - intact urine	volatile organic compounds	3	3/15	0	-
28	GCMS	Housing conditions - females - denatured urine	volatile organic compounds	6	2/15	1	Heptanone

Table S4.A. LME model for effects of social status and Enclosure phase on male urinary protein excretion (log transformed PC ratio)

FULL MODEL

Fixed effect	numDF	denDF	F value	p value
**(Intercept)**	**1**	**245**	**8.11**	**0.005**
**Social status**	**2**	**245**	**9.94**	**0.0001**
**Enclosure phase**	**2**	**245**	**4.02**	**0.019**
Age	1	245	0.27	0.60
Mass	1	245	0.63	0.43
**Social status*Enclosure phas**	**4**	**245**	**4.32**	**0.002**

POSTHOC TESTS

Factor: enclosure phase	Comparison	Estimate	Std. Error	z value	p value
Before enclosure	DOM vs SUB	0.020	0.186	0.106	1.00
	DOM vs CTRL	0.052	0.164	0.317	1.00
	SUB vs CTRL	0.032	0.145	0.223	1.00
During enclosure	**DOM vs SUB**	**0.792**	**0.214**	**3.706**	**0.006**
	**DOM vs CTRL**	**0.562**	**0.145**	**3.881**	**0.003**
	SUB vs CTRL	-0.230	0.194	-1.184	0.95
After enclosure	DOM vs SUB	0.216	0.342	-0.632	0.99
	DOM vs CTRL	-0.036	0.206	-0.173	1.00
	SUB vs CTRL	-0.252	0.309	-0.814	0.99

Factor: social status	Comparison	Estimate	Std. Error	z value	p value
Dominant males	**During vs before**	**0.762**	**0.146**	**5.204**	**< 0.001**
	After vs during	-0.223	0.183	-1.219	0.94
	After vs before	0.540	0.196	2.757	0.11
Subordinate males	During vs before	-0.011	0.195	-0.054	1.00
	After vs during	0.354	0.306	1.155	0.96
	After vs before	0.343	0.301	1.141	0.96
Cage control males	**During vs before**	**0.252**	**0.078**	**3.249**	**0.026**
	**After vs during**	**0.375**	**0.098**	**3.844**	**0.003**
	**After vs before**	**0.627**	**0.103**	**6.091**	**< 0.001**

Table S4.B. LME model for effects of social status and enclosure phase on male total urinary protein excretion

FULL MODEL

Fixed effect	numDF	denDF	F value	p value
**(Intercept)**	**1**	**245**	**24.35**	**<0.0001**
**Social status**	**2**	**245**	**5.19**	**0.006**
**Enclosure phase**	**2**	**245**	**30.63**	**<0.0001**
**Age**	**1**	**245**	**5.43**	**0.021**
Mass	1	245	0.01	0.91
**Social status*Enclosure phas**	**4**	**245**	**8.79**	**<0.0001**

POSTHOC TESTS

Factor: enclosure phase	Comparison	Estimate	Std. Error	z value	p value
Before enclosure	DOM vs SUB	0.235	0.246	0.956	0.99
	DOM vs CTRL	-0.468	0.238	-1.967	0.54
	**SUB vs CTRL**	**-0.703**	**0.195**	**-3.592**	**0.009**
During enclosure	DOM vs SUB	-0.088	0.273	-0.325	1.00
	DOM vs CTRL	0.577	0.203	2.839	0.09
	SUB vs CTRL	0.666	0.248	2.686	0.14
After enclosure	DOM vs SUB	-0.588	0.447	-1.314	0.92
	DOM vs CTRL	-0.730	0.313	-2.330	0.30
	SUB vs CTRL	-0.141	0.405	-0.350	0.99

Factor: social status	Comparison	Estimate	Std. Error	z value	p value
Dominant males	**During vs before**	**1.626**	**0.224**	**7.243**	**< 0.001**
	After vs during	0.033	0.279	0.119	1.00
	**After vs before**	**1.659**	**0.299**	**5.534**	**< 0.001**
Subordinate males	**During vs before**	**1.950**	**0.243**	**7.996**	**< 0.001**
	After vs during	0.532	0.388	1.371	0.90
	**After vs before**	**2.483**	**0.378**	**6.559**	**< 0.001**
Cage control males	**During vs before**	**0.580**	**0.151**	**3.842**	**0.003**
	**After vs during**	**1.341**	**0.190**	**7.045**	**< 0.001**
	**After vs before**	**1.921**	**0.200**	**9.571**	**< 0.001**

Table S4.C. LME model for effects of social status and enclosure phase on male creatinine excretion (log transformed)

FULL MODEL

Fixed effect	numDF	denDF	F value	p value
**(Intercept)**	**1**	**245**	**73.67**	**<0.0001**
**Social status**	**2**	**245**	**4.32**	**0.014**
Enclosure phase	2	245	0.23	0.80
Age	1	245	0.30	0.58
Mass	1	245	0.13	0.71
**Social status*Enclosure phase**	**4**	**245**	**2.75**	**0.029**

POSTHOC TESTS

Factor: enclosure phase	Comparison	Estimate	Std. Error	z value	p value
Before enclosure	DOM vs SUB	0.112	0.222	0.506	1.00
	DOM vs CTRL	-0.233	0.203	-1.144	0.96
	SUB vs CTRL	-0.345	0.172	-2.000	0.51
During enclosure	DOM vs SUB	-0.725	0.248	-2.922	0.07
	DOM vs CTRL	-0.362	0.181	-2.005	0.50
	SUB vs CTRL	0.363	0.224	1.618	0.77
After enclosure	DOM vs SUB	-0.358	0.389	-0.920	0.99
	DOM vs CTRL	-0.127	0.254	-0.499	1.00
	SUB vs CTRL	0.231	0.348	0.664	1.00

Factor: social status	Comparison	Estimate	Std. Error	z value	p value
Dominant males	During vs before	-0.108	0.177	-0.612	1.00
	After vs during	0.254	0.221	1.151	0.96
	After vs before	0.146	0.237	0.616	1.00
Subordinate males	**During vs before**	**0.729**	**0.217**	**3.366**	**0.019**
	After vs during	-0.113	0.333	-0.341	1.00
	After vs before	0.616	0.330	1.867	0.60
Cage control males	During vs before	0.021	0.099	0.215	1.00
	After vs during	0.018	0.125	0.148	1.00
	After vs before	0.040	0.132	0.302	1.00

Table S5.A. LME model for effects of social status and enclosure phase on female PC ratio (log transformed)

FULL MODEL

Fixed effect	numDF	denDF	F value	p value
**(Intercept)**	**1**	**288**	**4.41**	**0.037**
**Social status**	**2**	**288**	**3.10**	**0.046**
**Enclosure phase**	**2**	**288**	**11.75**	**<0.0001**
Age	1	288	0.004	0.95
Mass	1	288	0.11	0.74
**Social status*Enclosure phase**	**4**	**288**	**3.50**	**0.008**

POSTHOC TESTS

Factor: enclosure phase	Comparison	Estimate	Std. Error	z value	p value
Before enclosure	DOM vs SUB	0.164	0.204	0.805	0.99
	DOM vs CTRL	0.008	0.194	0.039	1.00
	SUB vs CTRL	-0.156	0.145	-1.081	0.97
During enclosure	DOM vs SUB	-0.208	0.171	-1.221	0.95
	DOM vs CTRL	0.119	0.163	0.733	0.99
	SUB vs CTRL	0.328	0.133	2.473	0.22
After enclosure	DOM vs SUB	0.059	0.270	0.218	1.00
	DOM vs CTRL	-0.196	0.248	-0.793	0.99
	SUB vs CTRL	-0.255	0.198	-1.289	0.93

Factor: social status	Comparison	Estimate	Std. Error	z value	p value
Dominant males	During vs before	0.134	0.186	0.724	0.99
	After vs during	0.329	0.231	1.425	0.87
	After vs before	0.463	0.246	1.887	0.59
Subordinate males	**During vs before**	**0.507**	**0.126**	**4.017**	**0.002**
	After vs during	0.062	0.169	0.366	0.99
	**After vs before**	**0.569**	**0.175**	**3.252**	**0.028**
Cage control males	During vs before	0.023	0.090	0.252	1.00
	**After vs during**	**0.645**	**0.115**	**5.586**	**< 0.001**
	**After vs before**	**0.667**	**0.122**	**5.460**	**< 0.001**

Table S5.B. LME model for effects of social status and enclosure phase on female total urinary protein production

FULL MODEL

Fixed effect	numDF	denDF	F value	p value
**(Intercept)**	**1**	**288**	**7.99**	**0.005**
**Social status**	**2**	**288**	**17.77**	**<0.0001**
**Enclosure phase**	**2**	**288**	**4.17**	**0.016**
Age	1	288	1.80	0.18
Mass	1	288	1.70	0.19
**Social status*Enclosure phase**	**4**	**288**	**10.45**	**<0.0001**

POSTHOC TESTS

Factor: enclosure phase	Comparison	Estimate	Std. Error	z value	p value
Before enclosure	DOM vs SUB	0.003	0.082	0.043	1.00
	DOM vs CTRL	-0.075	0.076	-0.987	0.99
	SUB vs CTRL	-0.079	0.055	-1.438	0.88
During enclosure	DOM vs SUB	-0.037	0.068	-0.553	0.99
	**DOM vs CTRL**	**0.246**	**0.064**	**3.842**	**<0.01**
	**SUB vs CTRL**	**0.283**	**0.050**	**5.635**	**<0.01**
After enclosure	DOM vs SUB	-0.001	0.110	-0.002	1.00
	DOM vs CTRL	0.106	0.099	1.074	0.97
	SUB vs CTRL	0.106	0.076	1.395	0.89

Factor: social status	Comparison	Estimate	Std. Error	z value	p value
Dominant males	**During vs before**	**0.336**	**0.077**	**4.314**	**<0.01**
	After vs during	-0.024	0.096	-0.249	1.00
	After vs before	0.312	0.103	3.025	0.06
Subordinate males	**During vs before**	**0.377**	**0.051**	**7.404**	**<0.01**
	After vs during	-0.061	0.068	-0.903	0.99
	**After vs before**	**0.315**	**0.070**	**4.466**	**<0.01**
Cage control males	During vs before	0.014	0.032	0.436	1.00
	After vs during	0.115	0.042	2.742	0.12
	After vs before	0.129	0.044	2.911	0.08

Table S5.C. LME model for effects of social status and enclosure phase on female creatinine excretion (log transformed)

FULL MODEL

Fixed effect	numDF	denDF	F value	p value
**(Intercept)**	**1**	**288**	**77.39**	**<0.0001**
Social status	2	288	1.15	0.32
Enclosure phase	2	288	1.63	0.20
Age	1	288	0.21	0.65
Mass	1	288	1.36	0.24
Social status*Enclosure phase	4	288	1.70	0.15

POSTHOC TESTS

Factor: Enclosure phase	Comparison	Estimate	Std. Error	z value	p value
Before enclosure	DOM vs SUB	-0.078	0.236	-0.331	1.00
	DOM vs CTRL	-0.143	0.272	-0.525	1.00
	SUB vs CTRL	-0.065	0.224	-0.289	1.00
During enclosure	DOM vs SUB	0.133	0.197	0.673	1.00
	DOM vs CTRL	0.409	0.242	1.690	0.72
	SUB vs CTRL	0.276	0.213	1.298	0.92
After enclosure	DOM vs SUB	-0.113	0.310	-0.363	1.00
	DOM vs CTRL	0.297	0.329	0.903	0.99
	SUB vs CTRL	0.410	0.275	1.493	0.84

Factor: social status	Comparison	Estimate	Std. Error	z value	p value
Dominant males	During vs before	0.470	0.215	2.186	0.38
	After vs during	-0.451	0.267	-1.685	0.72
	After vs before	0.020	0.284	0.069	1.00
Subordinate males	During vs before	0.259	0.138	1.871	0.60
	After vs during	-0.205	0.186	-1.103	0.97
	After vs before	0.054	0.192	0.282	1.00
Cage control males	During vs before	-0.082	0.121	-0.676	1.00
	After vs during	-0.339	0.155	-2.191	0.37
	After vs before	-0.421	0.164	-2.566	0.18

Table S6. Comparison of body mass parameters of dominant and subordinate mice (Mann-Whitney U tests)

Variable	Mean (SEM) of dominant mice (g)	Mean (SEM) of subordinate mice (g)	U	Sig. (2-tailed)
Female initial mass	21.1 (1.03)	20.1 (0.52)	104	0.55
Male initial mass	22.9 (0.69)	23.7 (1.15)	119	0.85
Male mean mass (during enclosure)	23.4 (0.41)	23.0 (0.50)	25	0.50
Male Δ mass (enclosure end - enclosure start)	0.27 (0.40)	-0.14 (0.75)	27	0.63

Table S7.A. GLMM results of sex and housing on urinary protein excretion.

**Model parameter**	**PC Ratio**			**Total Protein**			**Creatinine**		
	**X^2**	**p**		**X^2**	**p**		**X^2**	**p**	
Sex	67.8	**< 0.001**		392.6	**< 0.001**		22.4	**< 0.001**	
Housing	5.2	**0.02**		12.5	**< 0.001**		1.1	0.30	
Sex*Housing	2.3	0.13		28.1	**< 0.001**		2.0	0.16	

Table S7.B. Sexual dimorphism of urinary protein excretion under seminatural vs caged-control housing conditions.

**Housing**	**PC Ratio (Mean (SEM))**			**Total Protein mg/mL (Mean (SEM))**			**Creatinine mg/mL (Mean (SEM))**		
	**Male**	**Female**	**M:F ratio**	**Male**	**Female**	**M:F ratio**	**Male**	**Female**	**M:F ratio**
Caged-control	10.67 (0.76)	2.80 (0.24)	3.81	2.91 (0.10)	0.44 (0.03)	6.61	0.39 (0.03)	0.22 (0.02)	1.77
Seminatural	15.50 (1.57)	3.64 (0.27)	4.26	3.52 (0.15)	0.71 (0.03)	4.96	0.38 (0.04)	0.27 (0.02)	1.41

Table S8.A. Comparing behavioral preferences for urine and water in Y-maze to null expectations. Hypothesis testing performed using one sample t-test. Non-normal data tested using wilcoxon sign rank tests

Positive Control Experiment: (Δ=Urine-Water), parametric variables

Behavior	t	df	Sig. (2-tailed)	Sig. after correction	Mean Difference (s)	95% Confidence Interval of the Difference Lower	Upper
**ΔCumulative duration**	**3.04**	**14**	**0.009**	**0.033**	**20.41**	**6.00**	**34.81**
**ΔCumulative latency**	**-2.69**	**14**	**0.018**	**0.045**	**-44.69**	**-80.40**	**-8.99**
**ΔChamber duration**	**2.96**	**14**	**0.010**	**0.033**	**17.43**	**4.81**	**30.04**
ΔChamber latency	-1.96	13	0.072	0.120	-43.97	-92.45	4.51
ΔBranch duration	1.58	14	0.137	0.171	2.98	-1.07	7.02
ΔSniffing duration	-0.84	12	0.419	0.419	-2.17	-7.82	3.48
ΔSniffing latency	-0.96	12	0.354	0.393	-22.45	-73.17	28.28

nonparametric variables

Behavior	V	Sig. (2-tailed)	Sig. after correction	Median
**ΔCumulative visits**	**97**	**0.005**	**0.033**	**1**
**ΔChamber visits**	**68**	**0.024**	**0.048**	**2**
ΔSniffing visits	10	0.098	0.140	0.5

Table S8.B. Comparing behavioral preferences for male and female urine in Y-maze to null expectations.

Sex Discrimination: (Δ=Male-Female), parametric variables

Behavior	t	df	Sig. (2-tailed)	Sig. after correction	Mean Difference (s)	95% Confidence Interval of the Difference Lower	Upper
ΔCumulative duration	0.81	9	0.440	0.957	5.52	-9.94	20.98
**ΔCumulative latency**	**-3.91**	**9**	**0.004**	**0.040**	**-40.11**	**-63.30**	**-16.93**
ΔChamber duration	0.93	9	0.379	0.957	5.74	-8.29	19.77
ΔChamber latency	-0.06	9	0.957	0.957	-1.53	-64.24	61.18
ΔBranch duration	-0.10	9	0.925	0.957	-0.22	-5.44	4.99
ΔSniffing duration	0.61	8	0.559	0.957	0.93	-2.60	4.47
ΔSniffing latency	0.11	8	0.917	0.957	3.57	-73.26	80.39

nonparametric variables

Behavior	V	Sig. (2-tailed)	Sig. after correction	Median
ΔCumulative visits	30	0.836	0.957	0
ΔChamber visits	33	0.607	0.957	1
ΔSniffing visits	27	0.633	0.957	1

Table S8.C. Comparing behavioral preferences for high and low urinary protein concentration in Y-maze to null expectations.

Concentration Discrimination: (Δ=High-Low) PC ratio, parametric variables

Behavior	t	df	Sig. (2-tailed)	Sig. after correction	Mean Difference (s)	95% Confidence Interval of the Difference Lower	Upper
ΔCumulative duration	-1.43	9	0.188	0.403	-13.44	-34.76	7.88
ΔCumulative latency	1.07	9	0.311	0.403	12.65	-14.01	39.30
ΔChamber duration	-	-	-	-	-	-	-
ΔChamber latency	1.63	9	0.137	0.403	21.22	-8.17	50.62
ΔBranch duration	-2.02	9	0.074	0.370	-3.94	-8.35	0.47
ΔSniffing duration	-0.07	8	0.948	0.964	-0.09	-3.05	2.88
ΔSniffing latency	0.41	8	0.694	0.964	8.86	-41.26	58.99

nonparametric variables

Behavior	V	Sig. (2-tailed)	Sig. after correction	Median
ΔCumulative visits	5	0.042	0.333	-1.5
ΔChamber visits	10.5	0.285	0.948	-0.5
ΔSniffing visits	21	0.265	0.333	1
ΔChamber duration (s)	17	0.322	0.362	-4.04

Table S9. LME model for effect of female estrous phase on latency to investigate dominant urine. Delta (Δ) indicates the response to dominant stimuli minus subordinate observed at the given variable.

Variable (Δ=DOM-SUB)	Diestrus mean (SEM)	Estrus mean (SEM)	Sum Sq	NumDF	DenDF	F value	Estimate	Std.Error	p value	adj. p-value
ΔCumulative duration (s)	-4.5 (13.3)	-13.7 (17.9)	422.46	1	9	0.25	-9.19	18.32	0.63	0.84
**ΔCumulative latency (ln transformed [s])**	**1.28 (0.8)**	**-2.72 (0.7)**	**80.12**	**1**	**4.0856**	**53.05**	**-4**	**0.55**	**0.00**	**0.02**
ΔChamber duration (ln transformed [s])	-0.95 (1.1)	-1.22 (1.14)	0.074	1	7.4855	0.01	-0.13	1.28	0.92	0.92
ΔChamber latency (ln transformed [s])	2.42 (0.6)	-2.38 (0.8)	92.5	1	1.2266	51.55	-4.94	0.69	0.06	0.3
ΔBranch duration (ln transformed [s])	0.33 (0.6)	-0.03 (0.6)	0.66	1	9	0.19	-0.36	0.83	0.67	0.84
ΔSniffing duration (ln transformed [s])	-0.60 (0.4)	-0.23 (0.4)	0.58	1	15	0.38	0.37	0.6	0.55	0.84
ΔSniffing latency (s)	-22.0 (13.1)	-19.9 (12.4)	0.0028	1	14.024	0.25	-1.57	3.12	0.62	0.84
ΔCumulative frequency	0.1 (0.5)	0.4 (0.5)	0.45	1	9	0.27	0.3	0.58	0.62	0.84
ΔChamber frequency	-0.9 (0.6)	0.1 (0.3)	5	1	18	2.15	1	0.68	0.16	0.53
ΔSniffing frequency	-0.1 (1.1)	-0.3 (0.62)	0.12	1	7.86	0.02	-0.17	1.23	0.90	0.92

POST HOC TESTS on Cumulative latency (ln [s])

**Factor: Estrous phase**	**Comparison**	**Estimate**	**std error**	**z value**	**p value**
**Estrus**	**SUB - DOM**	**1.03547**	**0.30046**	**3.446**	**0.00318**
Diestrus	SUB - DOM	-0.56025	0.30046	-1.865	0.24327
**Estrous Phase * Urine Donor Social Status [Estrus (E) - Diestrus (D)]**	**SUB (E) - SUB (D)**	**0.97413**	**0.30046**	**3.242**	**0.00651**
	DOM (E) - DOM (D)	-0.62158	0.30046	-2.069	0.16323
	SUB (E) - DOM (D)	0.41389	0.30046	1.378	0.5135
	DOM (E) - SUB (D)	-0.06133	0.30046	0.204	0.99699

Table S10.A. Predicting social status from relative intensity of MUPs before enclosure

**Protein**	**Mean (SD) of prospective dominant males**	**Mean (SD) of prospective subordinate males**	**Benjamini-Hochberg corrected p-value**	**Fold change**
MUP1	0.008 (0.004)	0.013 (0.017)	0.8041	1.63
MUP4	0.072 (0.035)	0.112 (0.033)	0.1054	1.56
MUP20	0.040 (0.015)	0.041 (0.027)	0.9714	1.02
MUP3	0.006 (0.005)	0.006 (0.004)	0.9714	1.00
MUP2	3.76E-04 (1.7E-04)	3.03E-04 (1.4E-04)	0.7660	-1.24
MUP5	0.093 (0.021)	0.066 (0.020)	0.0950	-1.41
MUP17	0.056 (0.014)	0.038 (0.016)	0.1054	-1.47

Table S10.B Comparing relative intensity of non-MUP proteins between social status groups during enclosure

**Protein**	**Mean (SD) of dominant males**	**Mean (SD) of subordinate males**	**Benjamini-Hochberg corrected p-value**	**Fold change**
Acyl-coenzyme A synthetase	0.118 (0.081)	0.460 (0.498)	0.9567	3.90
Electron transfer flavoprotein subunit beta	0.058 (0.064)	0.107 (0.057)	0.9567	1.84
UPF0500 protein C1orf216 homolog	4.635 (1.587)	2.155 (1.143)	0.9567	-2.15

Table S10.C. Comparing differences in MUP protein excretion between social status groups

**Name**	**Mean (SD) of dominant males**	**Mean (SD) of subordinate males**	**Benjamini-Hochberg corrected p-value**	**Fold change**
**Mup2**	0.336 (0.160)	0.849 (0.465)	0.0001	2.53
**Mup1**	1.416 (0.422)	1.783 (0.233)	0.0073	1.26
**Mup20**	2.101 (0.303)	2.481 (0.538)	0.0041	1.18
Mup3	1.187 (0.460)	1.401 (0.504)	0.2181	1.18
**Mup17**	2.228 (0.222)	2.564 (0.402)	0.0004	1.15
**Mup5**	2.481 (0.202)	2.769 (0.161)	0.0003	1.12
**Mup4**	2.443 (0.302)	2.718 (0.588)	0.0070	1.11

Table S10.D. Comparing differences in non-MUP protein relative intensity between social status groups

**Name**	**Mean (SD) of dominant males**	**Mean (SD) of subordinate males**	**Benjamini-Hochberg corrected p-value**	**fold change**
**P23953_EST1C_MOUSE**	0.178 (0.073)	0.471 (0.301)	<0.001	2.64
**P51437_CRAMP_MOUSE**	0.040 (0.032)	0.145 (0.084)	<0.001	3.64
**Q9CZ13_QCR1_MOUSE**	0.013 (0.004)	0.081 (0.194)	<0.001	6.13
**P21614_VTDB_MOUSE**	0.119 (0.096)	0.360 (0.293)	<0.001	3.03
**Q8BP99_CA216_MOUSE**	1.221 (0.551)	3.906 (1.854)	<0.001	3.20
**Q00898_A1AT5_MOUSE**	0.769 (1.694)	4.047 (2.930)	<0.001	5.27
**Q60590_A1AG1_MOUSE**	0.192 (0.128)	0.557 (0.292)	0.001	2.90
**P04104_K2C1_MOUSE**	0.007 (0.003)	0.027 (0.015)	0.001	4.02
**P20918_PLMN_MOUSE**	0.150 (0.114)	0.518 (0.365)	0.0056	3.44
**P56391_CX6B1_MOUSE**	0.033 (0.023)	0.222 (0.308)	0.0062	6.77
**P28665_MUG1_MOUSE**	0.224 (0.241)	0.615 (0.596)	0.0068	2.74
**P12246_SAMP_MOUSE**	0.047 (0.045)	0.107 (0.061)	0.0096	2.29
**P0CW02_LY6C1_MOUSE**	0.138 (0.096)	0.173 (0.176)	0.0104	1.25
**Q9DB77_QCR2_MOUSE**	0.017 (0.014)	0.082 (0.062)	0.0118	4.75
**A6X935_ITIH4_MOUSE**	0.087 (0.192)	0.137 (0.131)	0.0174	1.58
**P11087_CO1A1_MOUSE**	0.103 (0.121)	0.291 (0.282)	0.0185	2.83
**Q8K0L3_ACSM2_MOUSE**	0.071 (0.024)	0.219 (0.319)	0.0196	3.08
**Q61147_CERU_MOUSE**	0.070 (0.109)	0.180 (0.380)	0.0233	2.58
**Q05920_PYC_MOUSE**	0.072 (0.045)	0.192 (0.300)	0.0233	2.67
**Q9QXC1_FETUB_MOUSE**	0.183 (0.144)	0.350 (0.191)	0.0233	1.92
**P09803_CADH1_MOUSE**	0.146 (0.089)	0.295 (0.141)	0.0233	2.02
**P63260_ACTG_MOUSE**	0.139 (0.092)	0.298 (0.199)	0.0235	2.15
**Q6IFZ6_K2C1B_MOUSE**	1.240 (0.787)	2.845 (2.648)	0.0269	2.29
**P01942_HBA_MOUSE**	0.240 (0.237)	0.805 (1.052)	0.029	3.36

O08677_KNG1_MOUSE	1.035 (0.948)	2.256 (1.636)	0.0313	2.18
P04186_CFAB_MOUSE	0.152 (0.181)	0.263 (0.194)	0.0468	1.74

Table S10.E. Comparing differences in non-MUP protein excretion between social status groups

Name	Mean (SD) of dominant males	Mean (SD) of subordinate males	Benjamini-Hochberg corrected p-value	fold change
A6X935_ITIH4_MOUSE	0.089 (0.155)	0.310 (0.190)	<0.001	3.47
O08677_KNG1_MOUSE	0.589 (0.240)	1.197 (0.446)	<0.001	2.03
O70570_PIGR_MOUSE	0.076 (0.091)	0.187 (0.105)	<0.001	2.45
P00688_AMYP_MOUSE	0.334 (0.224)	0.821 (0.340)	<0.001	2.45
P01027_CO3_MOUSE	0.279 (0.164)	0.704 (0.272)	<0.001	2.53
P01837_IGKC_MOUSE	0.201 (0.111)	0.568 (0.257)	<0.001	2.83
P01942_HBA_MOUSE	0.237 (0.230)	0.749 (0.368)	<0.001	3.16
P02088_HBB1_MOUSE	0.224 (0.131)	0.505 (0.241)	<0.001	2.26
P02089_HBB2_MOUSE	0.130 (0.070)	0.376 (0.250)	<0.001	2.90
P02535_K1C10_MOUSE	0.830 (0.272)	1.193 (0.202)	<0.001	1.44
P02816_PIP_MOUSE	0.218 (0.159)	0.539 (0.307)	<0.001	2.47
P03953_CFAD_MOUSE	1.525 (0.325)	1.895 (0.205)	<0.001	1.24
P04104_K2C1_MOUSE	0.011 (0.009)	0.089 (0.053)	<0.001	8.36
P04186_CFAB_MOUSE	0.177 (0.198)	0.481 (0.224)	<0.001	2.72
P05533_LY6A_MOUSE	0.338 (0.138)	0.693 (0.225)	<0.001	2.05
P07724_ALBU_MOUSE	1.101 (0.197)	1.570 (0.480)	<0.001	1.43
P07759_SPA3K_MOUSE	0.249 (0.224)	0.631 (0.392)	<0.001	2.53
P09803_CADH1_MOUSE	0.165 (0.166)	0.471 (0.221)	<0.001	2.86
P0CW02_LY6C1_MOUSE	0.183 (0.133)	0.536 (0.182)	<0.001	2.92
P11087_CO1A1_MOUSE	0.135 (0.150)	0.469 (0.270)	<0.001	3.47
P12246_SAMP_MOUSE	0.069 (0.073)	0.286 (0.140)	<0.001	4.11
P19001_K1C19_MOUSE	0.186 (0.128)	0.463 (0.238)	<0.001	2.49
P20918_PLMN_MOUSE	0.179 (0.124)	0.690 (0.288)	<0.001	3.85
P21614_VTDB_MOUSE	0.153 (0.122)	0.576 (0.246)	<0.001	3.77
P23953_EST1C_MOUSE	0.214 (0.110)	0.674 (0.227)	<0.001	3.15
P28665_MUG1_MOUSE	0.226 (0.173)	0.714 (0.294)	<0.001	3.16
P29788_VTNC_MOUSE	0.125 (0.136)	0.459 (0.321)	<0.001	3.68
P35459_LY6D_MOUSE	0.666 (0.249)	1.044 (0.243)	<0.001	1.57
P45952_ACADM_MOUSE	0.234 (0.155)	0.562 (0.261)	<0.001	2.41
P50446_K2C6A_MOUSE	0.176 (0.062)	0.453 (0.455)	<0.001	2.58
P51437_CRAMP_MOUSE	0.062 (0.056)	0.339 (0.172)	<0.001	5.47
P54071_IDHP_MOUSE	0.044 (0.052)	0.124 (0.068)	<0.001	2.79
P56391_CX6B1_MOUSE	0.053 (0.059)	0.390 (0.271)	<0.001	7.32
P56480_ATPB_MOUSE	0.299 (0.111)	0.743 (0.308)	<0.001	2.48
P63260_ACTG_MOUSE	0.173 (0.119)	0.523 (0.225)	<0.001	3.02
P70269_CATE_MOUSE	0.569 (0.281)	1.040 (0.319)	<0.001	1.83
Q00623_APOA1_MOUSE	0.052 (0.047)	0.215 (0.193)	<0.001	4.11
Q00898_A1AT5_MOUSE	0.392 (0.351)	1.457 (0.275)	<0.001	3.72
Q02257_PLAK_MOUSE	0.119 (0.093)	0.408 (0.252)	<0.001	3.41
Q05793_PGBM_MOUSE	0.026 (0.020)	0.095 (0.111)	<0.001	3.67
Q05920_PYC_MOUSE	0.092 (0.116)	0.302 (0.266)	<0.001	3.28
Q07456_AMBP_MOUSE	0.687 (0.316)	1.212 (0.261)	<0.001	1.76
Q3TTY5_K22E_MOUSE	0.534 (0.132)	0.782 (0.150)	<0.001	1.46
Q497I4_KRT35_MOUSE	0.171 (0.125)	0.527 (0.268)	<0.001	3.08
Q60590_A1AG1_MOUSE	0.217 (0.140)	0.725 (0.246)	<0.001	3.34
Q60928_GGT1_MOUSE	0.131 (0.103)	0.398 (0.203)	<0.001	3.05
Q60932_VDAC1_MOUSE	0.353 (0.195)	0.705 (0.304)	<0.001	2.00
Q61147_CERU_MOUSE	0.212 (0.177)	0.587 (0.212)	<0.001	2.77
Q61207_SAP_MOUSE	1.041 (0.176)	1.368 (0.233)	<0.001	1.31
Q61414_K1C15_MOUSE	0.339 (0.160)	0.696 (0.129)	<0.001	2.05
Q61646_HPT_MOUSE	0.746 (0.270)	1.270 (0.409)	<0.001	1.70
Q61838_A2M_MOUSE	0.374 (0.184)	0.827 (0.342)	<0.001	2.21
Q6IFZ6_K2C1B_MOUSE	0.665 (0.187)	1.291 (0.263)	<0.001	1.94

**Q6NXH9_K2C73_MOUSE**	0.725 (0.221)	1.208 (0.204)	<0.001	1.67
**Q8BHC0_LYVE1_MOUSE**	0.447 (0.200)	0.875 (0.200)	<0.001	1.96
**Q8BP99_CA216_MOUSE**	0.681 (0.209)	1.490 (0.320)	<0.001	2.19
**Q8C6C9_CF058_MOUSE**	0.624 (0.210)	1.081 (0.318)	<0.001	1.73
**Q8K0L3_ACSM2_MOUSE**	0.101 (0.057)	0.378 (0.272)	<0.001	3.75
**Q921I1_TRFE_MOUSE**	0.521 (0.142)	0.919 (0.430)	<0.001	1.77
**Q922U2_K2C5_MOUSE**	0.215 (0.164)	0.438 (0.226)	<0.001	2.04
**Q9CZ13_QCR1_MOUSE**	0.021 (0.010)	0.160 (0.218)	<0.001	7.77
**Q9DAU7_WFDC2_MOUSE**	0.185 (0.086)	0.417 (0.251)	<0.001	2.25
**Q9DB77_QCR2_MOUSE**	0.031 (0.042)	0.223 (0.147)	<0.001	7.24
**Q9DBD0_ICA_MOUSE**	0.125 (0.074)	0.288 (0.110)	<0.001	2.30
**Q9EQ20_MMSA_MOUSE**	0.335 (0.147)	0.682 (0.221)	<0.001	2.03
**Q9QWL7_K1C17_MOUSE**	0.248 (0.113)	0.633 (0.326)	<0.001	2.55
**Q9QXC1_FETUB_MOUSE**	0.096 (0.063)	0.344 (0.246)	<0.001	3.57
**Q9Z2K1_K1C16_MOUSE**	0.205 (0.054)	0.551 (0.297)	<0.001	2.69
**O08997_ATOX1_MOUSE**	0.075 (0.070)	0.193 (0.112)	0.0011	2.57
**O89020_AFAM_MOUSE**	0.050 (0.063)	0.205 (0.154)	0.0011	4.10
**Q99KI0_ACON_MOUSE**	0.265 (0.248)	0.567 (0.243)	0.0011	2.14
**P28798_GRN_MOUSE**	0.338 (0.176)	0.557 (0.179)	0.0013	1.65
**Q9DBM2_ECHP_MOUSE**	0.038 (0.041)	0.105 (0.087)	0.0013	2.75
**P61110_ANRE_MOUSE**	0.660 (0.386)	1.171 (0.420)	0.0017	1.78
**P01869_IGH1M_MOUSE**	0.333 (0.269)	0.770 (0.428)	0.0018	2.31
**P22599_A1AT2_MOUSE**	0.087 (0.037)	0.242 (0.188)	0.0023	2.76
**P15947_KLK1_MOUSE**	1.043 (0.358)	1.442 (0.300)	0.0026	1.38
**Q8VDN2_AT1A1_MOUSE**	0.274 (0.151)	0.515 (0.240)	0.0026	1.88
**P51910_APOD_MOUSE**	0.273 (0.162)	0.526 (0.256)	0.0031	1.93
**P07758_A1AT1_MOUSE**	0.395 (0.302)	0.981 (0.508)	0.0033	2.48
**Q3UV17_K22O_MOUSE**	0.070 (0.039)	0.187 (0.219)	0.0035	2.68
**P01843_LAC1_MOUSE**	0.207 (0.176)	0.490 (0.302)	0.0045	2.36
**P01867_IGG2B_MOUSE**	0.113 (0.074)	0.253 (0.215)	0.0045	2.23
**P08228_SODC_MOUSE**	0.335 (0.352)	0.678 (0.281)	0.0045	2.03
**Q91X17_UROM_MOUSE**	1.193 (0.226)	1.449 (0.230)	0.0045	1.21
**Q8BZH1_TGM4_MOUSE**	0.143 (0.135)	0.379 (0.238)	0.0046	2.65
**Q9Z0K8_VNN1_MOUSE**	0.079 (0.095)	0.233 (0.197)	0.0053	2.94
**Q03265_ATPA_MOUSE**	0.160 (0.126)	0.410 (0.322)	0.0062	2.57
**Q6IFX2_K1C42_MOUSE**	0.508 (0.322)	0.911 (0.412)	0.0062	1.79
**Q00897_A1AT4_MOUSE**	0.215 (0.150)	0.416 (0.221)	0.0067	1.93
**P10923_OSTP_MOUSE**	0.153 (0.087)	0.327 (0.219)	0.0068	2.14
**Q62395_TFF3_MOUSE**	0.322 (0.219)	0.643 (0.368)	0.0074	1.99
**A2ARV4_LRP2_MOUSE**	0.245 (0.199)	0.512 (0.333)	0.0075	2.09
**O09114_PTGDS_MOUSE**	0.285 (0.127)	0.547 (0.389)	0.0081	1.92
**P01865_GCAM_MOUSE**	0.042 (0.052)	0.109 (0.089)	0.0081	2.59
**P63038_CH60_MOUSE**	0.203 (0.185)	0.377 (0.203)	0.0081	1.85
**Q06890_CLUS_MOUSE**	0.433 (0.317)	0.723 (0.244)	0.0081	1.67
**P48962_ADT1_MOUSE**	0.142 (0.190)	0.305 (0.228)	0.0089	2.15
**P29699_FETUA_MOUSE**	0.460 (0.402)	0.705 (0.337)	0.0099	1.53
**Q6NZJ6_IF4G1_MOUSE**	0.506 (0.709)	1.034 (1.052)	0.0122	2.04
**P28825_MEP1A_MOUSE**	0.818 (0.362)	1.167 (0.371)	0.0125	1.43
**Q03401_CRIS1_MOUSE**	0.103 (0.103)	0.178 (0.103)	0.02	1.72
**Q9DCW4_ETFB_MOUSE**	0.092 (0.077)	0.199 (0.154)	0.0214	2.18
**P09036_ISK3_MOUSE**	0.217 (0.229)	0.287 (0.132)	0.0317	1.32
**Q91X72_HEMO_MOUSE**	0.401 (0.234)	0.615 (0.302)	0.0358	1.53
**P49183_DNAS1_MOUSE**	1.206 (0.249)	1.415 (0.299)	0.0433	1.17

Table S11.A. Comparing total protein fold change in dominant male urinary excretion during group housing to null expectations.

						95% Confidence Interval of the Difference	
**protein amount**	**t**	**df**	**Sig. (2-tailed)**	**Sig. after correction**	**Mean Difference**	**Lower**	**Upper**
**Mup20**	5.62	11	0.000	0.003	0.567	0.345	0.788
**Mup17**	3.85	11	0.003	0.007	1.522	0.651	2.393
Mup3	1.51	11	0.158	0.184	0.291	-0.132	0.715
Mup1	2.33	11	0.040	0.056	0.389	0.022	0.756
**Mup5**	2.85	11	0.016	0.028	0.218	0.049	0.386
**Mup2**	4.32	11	0.001	0.004	0.779	0.382	1.176
Mup4	1.37	11	0.197	0.197	0.299	-0.180	0.777

Table S11.B. Comparing total protein fold change in subordinate male urinary excretion during group housing to null expectations.

						95% Confidence Interval of the Difference	
**protein amount**	**t**	**df**	**Sig. (2-tailed)**	**Sig. after correction**	**Mean Difference**	**Lower**	**Upper**
Mup20	1.25	4	0.281	0.281	1.193	-1.464	3.849
Mup17	2.45	4	0.071	0.166	3.327	-0.449	7.104
Mup3	1.83	4	0.141	0.197	1.822	-0.942	4.586
Mup1	1.86	4	0.136	0.197	3.735	-1.830	9.299
Mup5	3.58	4	0.023	0.161	2.134	0.478	3.790
Mup2	2.47	4	0.069	0.166	4.280	-0.535	9.095
Mup4	1.33	4	0.256	0.281	2.736	-2.995	8.468

Table S11.C. Comparing relative intensity fold change in dominant male urinary protein excretion during group housing to null expectations.

						95% Confidence Interval of the Difference	
**relative intensity**	**t**	**df**	**Sig. (2-tailed)**	**Sig. after correction**	**Mean Difference**	**Lower**	**Upper**
Mup20	2.35	11	0.038	0.089	0.599	0.039	1.158
Mup17	-0.36	11	0.726	0.821	-0.047	-0.337	0.242
Mup3	-0.75	11	0.471	0.660	-0.127	-0.503	0.248
Mup1	-0.23	11	0.821	0.821	-0.030	-0.313	0.253
**Mup5**	-6.17	11	0.000	0.003	-0.348	-0.472	-0.224
Mup2	2.39	11	0.036	0.089	0.360	0.029	0.692
Mup4	1.25	11	0.237	0.415	0.515	-0.392	1.423

Table S11.D. Comparing relative intensity fold change in subordinate male urinary protein excretion during group housing to null expectations.

						95% Confidence Interval of the Difference	
**relative intensity**	**t**	**df**	**Sig. (2-tailed)**	**Sig. after correction**	**Mean Difference**	**Lower**	**Upper**
Mup20	-1.50	4	0.208	0.525	-0.372	-1.061	0.317
Mup17	1.00	4	0.375	0.525	0.363	-0.648	1.375
Mup3	-0.79	4	0.471	0.550	-0.197	-0.887	0.492
Mup1	1.16	4	0.309	0.525	0.539	-0.747	1.826
Mup5	1.04	4	0.358	0.525	0.389	-0.651	1.429
Mup2	1.85	4	0.138	0.525	0.634	-0.317	1.584
Mup4	0.14	4	0.898	0.900	0.072	-1.389	1.532

Regulation of volatile and non-volatile pheromone attractants depends upon male social status

Supplementary Figures S1 Fig. 1 to S1 Fig. 12

Regulation of volatile and non-volatile pheromone attractants depends upon male social status

S1. Supplemental Figures

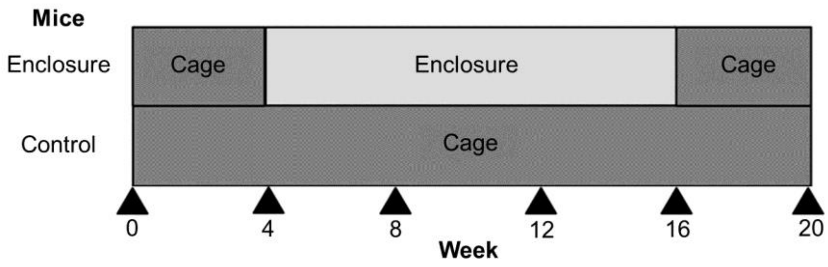

S1 Fig 1. Urine collection regime.

Urine collections (indicated by black triangle) were collected in 4 week intervals. Mice were housed singly in cages except for mice housed in seminatural enclosures during the 12 week enclosure phase.

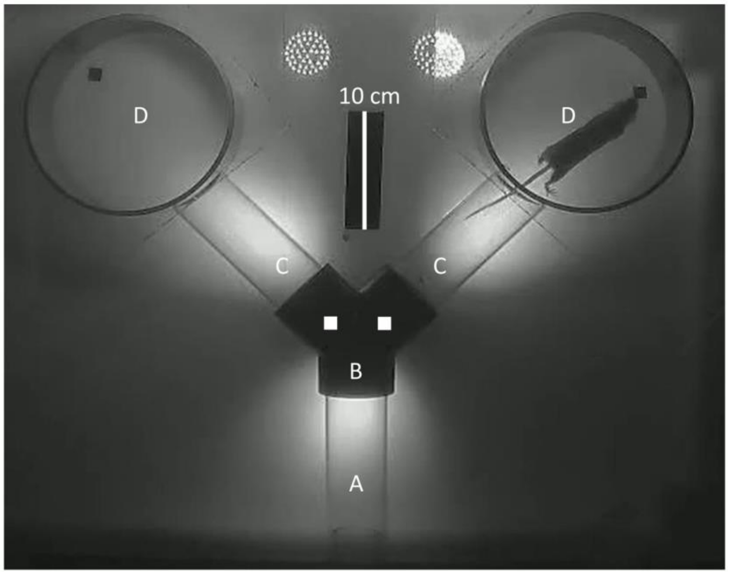

S1 Fig 2. Y-maze apparatus with test mouse for olfactory discrimination.

A) Primary branch (20 cm), B) Decision zone, C) Secondary branches (15 cm), D) Chambers (16 cm Ø and 8 cm height) secured by plexiglass planes. Competing test stimuli were placed on the black and white squares. Backlighting and overhead lighting (note reflection at top of image) provided by 48-LED infrared lights.

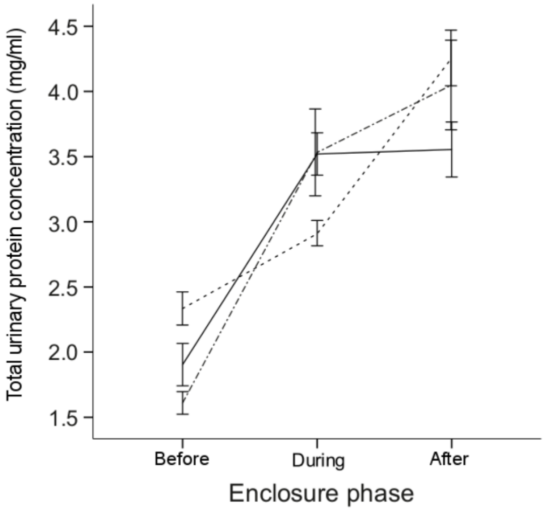

S1 Fig 3. Male total urinary protein excretion increases over time.

Total urinary protein excretion before, during, and after housing in seminatural enclosures for socially dominant (solid line) and subordinate (dot-dashed line) males compared to caged control males (dashed line). Error bars indicate mean ± 1 s.e.m.

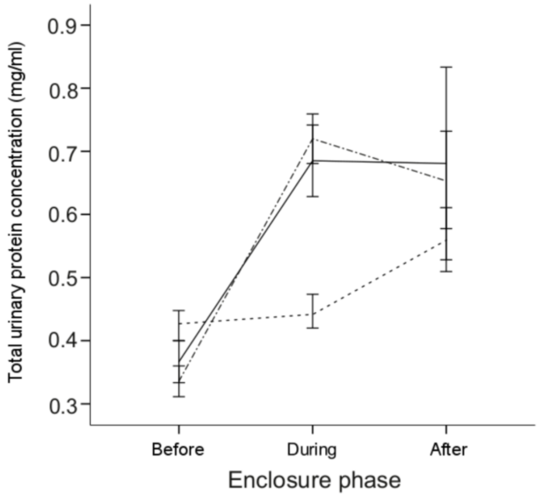

S1 Fig 4. Enclosure females upregulate total urinary protein excretion during seminatural conditions.

Total urinary protein excretion before, during, and after housing in semi-natural enclosures for socially dominant (solid line) and subordinate (dot-dashed line) females compared to caged control females (dashed line). Error bars indicate mean ± 1 s.e.m.

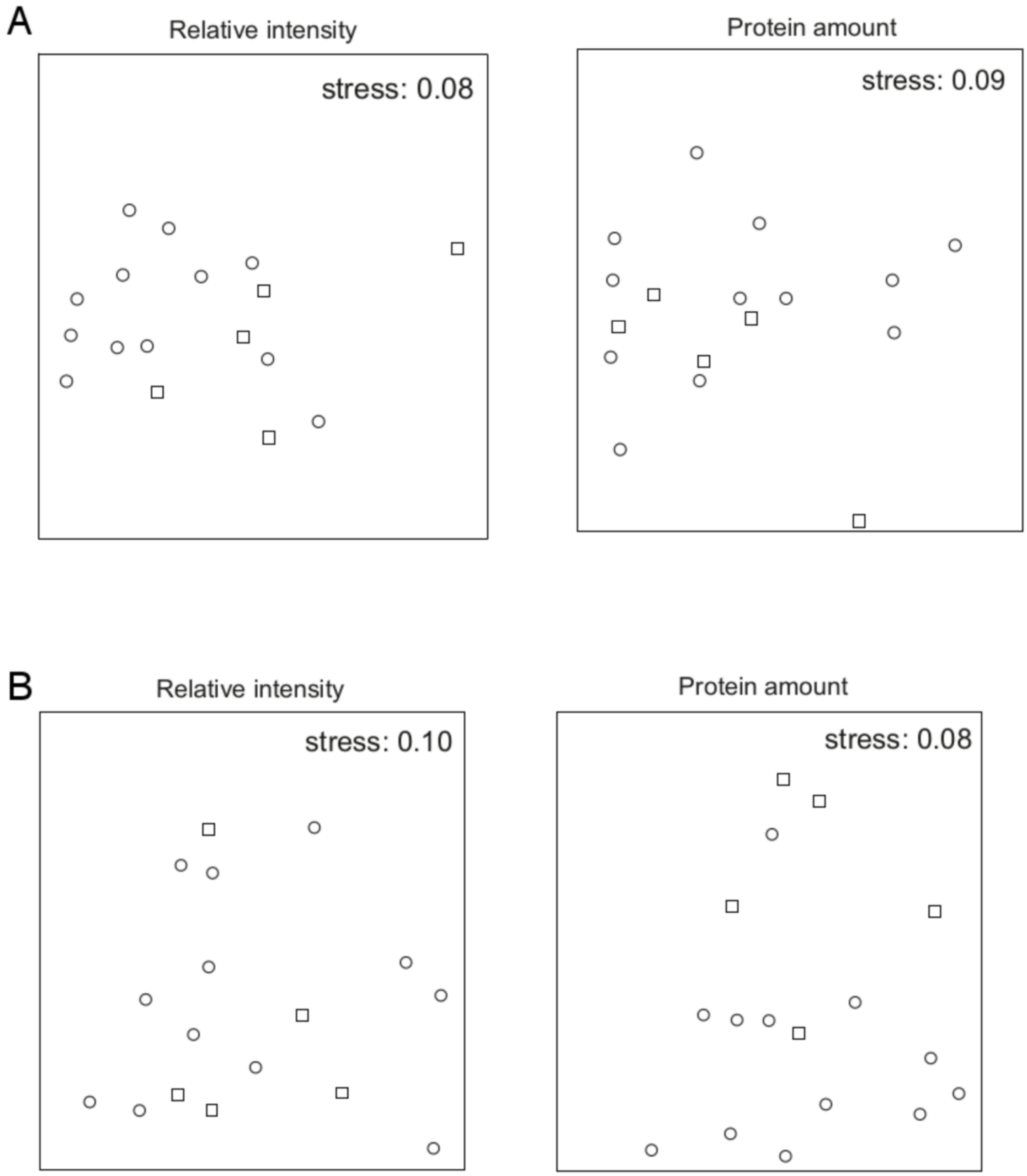

S1 Fig 5. Urinary protein profile similarity between males before release into seminatural conditions.

Protein profile similarity of males that later became dominant (circles) and subordinate (squares) before the enclosure phase visualized using nonmetric multidimensional scaling. Data points closer together represent individuals with relatively high chemical similarity in relative intensity (left side) or protein amount (right side) of (A) MUPs and (B) non-MUP urinary proteins.

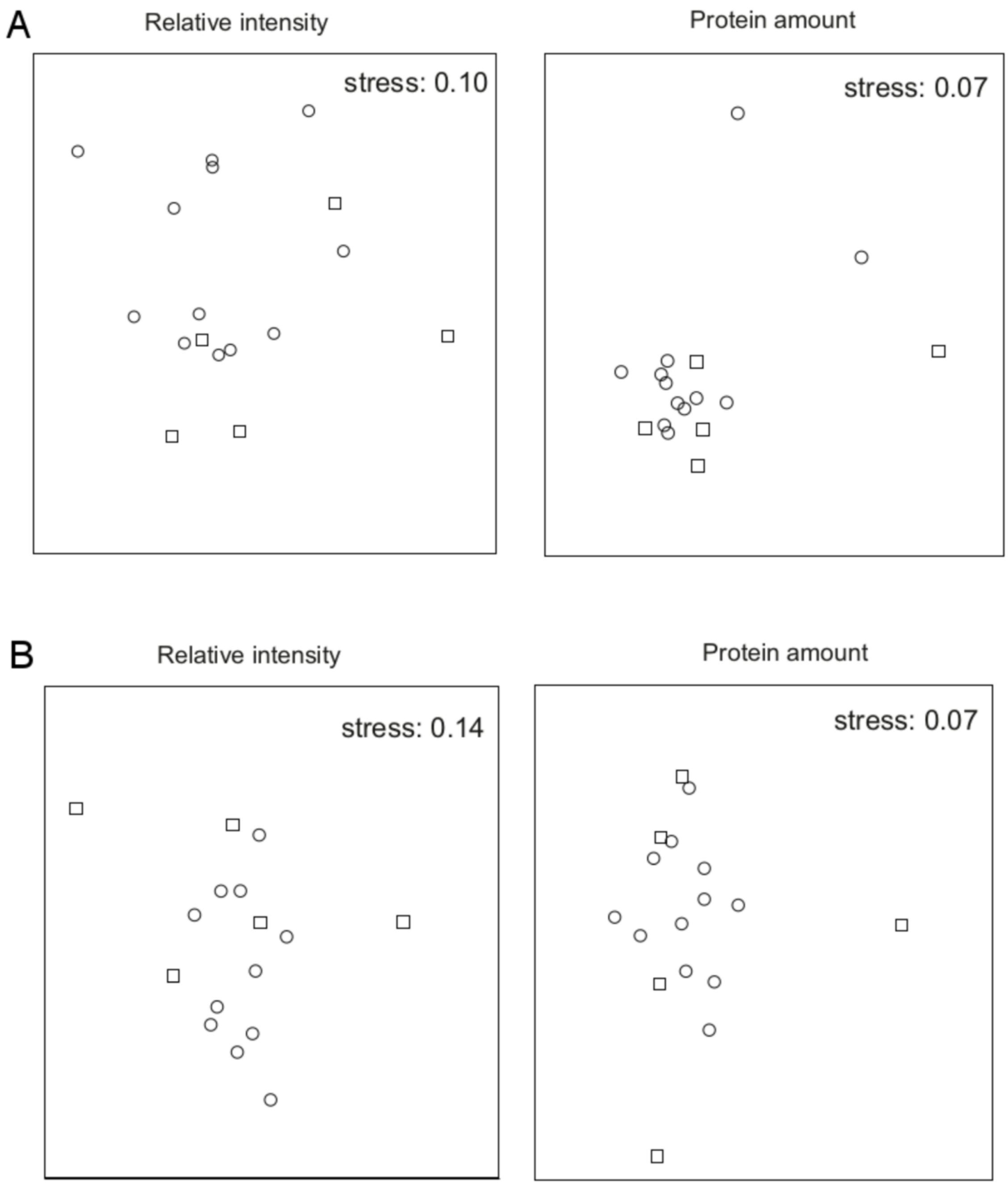

S1 Fig 6. Urinary protein profile similarity between males during seminatural conditions.

Protein profile similarity of dominant (circles) and subordinate (squares) males during the enclosure phase visualized using nonmetric multidimensional scaling. Data points closer together represent individuals with relatively high chemical similarity in relative intensity (left side) or protein amount (right side) of (A) MUPs and (B) non-MUP urinary proteins.

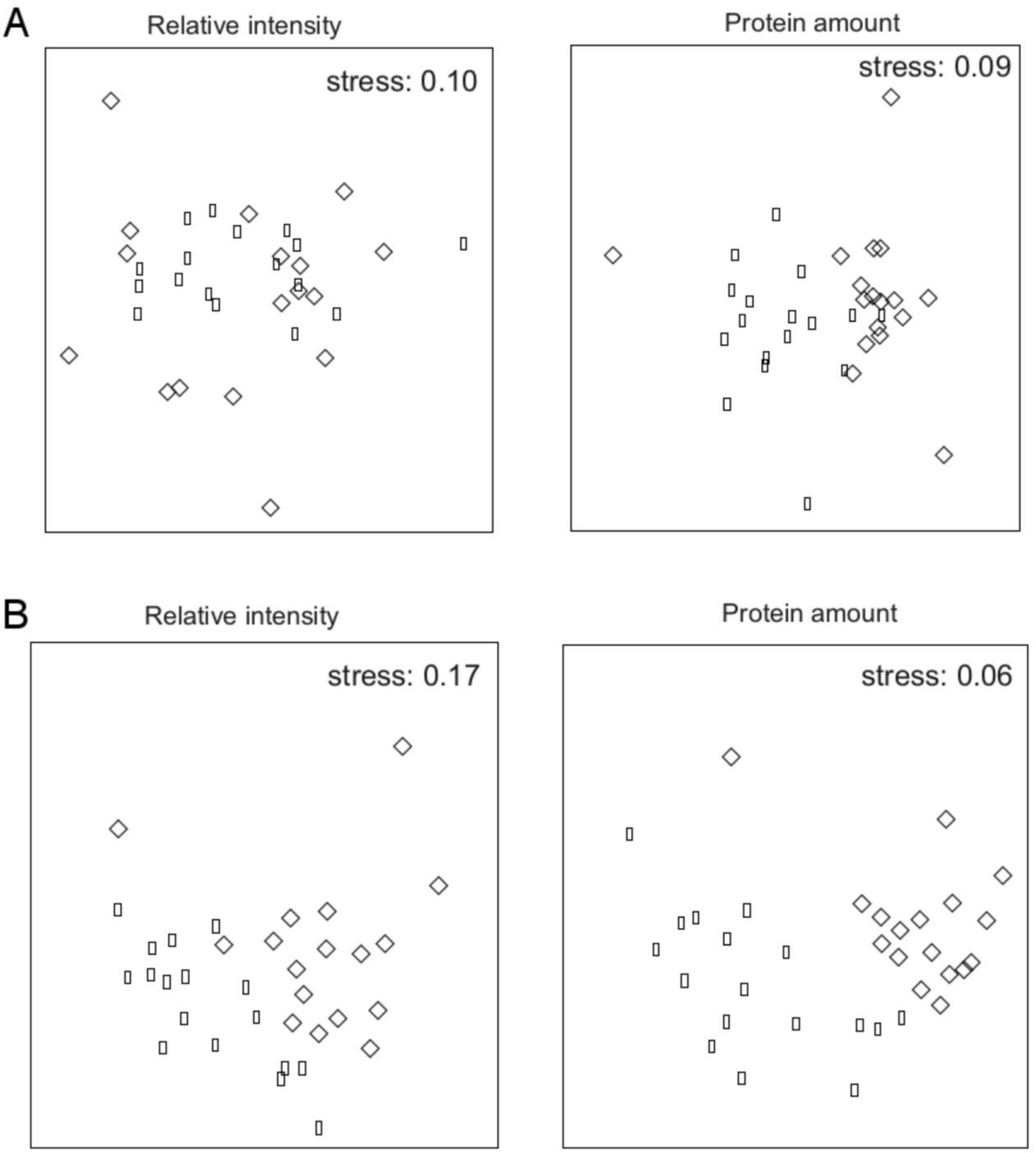

S1 Fig 7. Effects of seminatural conditions on urinary protein profile similarity within males.

Protein profile similarity of males before (rectangle) and during (rhombus) the enclosure phase visualized using nonmetric multidimensional scaling. Data points closer together represent individuals with relatively high chemical similarity in relative intensity (left side) or protein amount (right side) of (A) MUPs and (B) non-MUP urinary proteins.

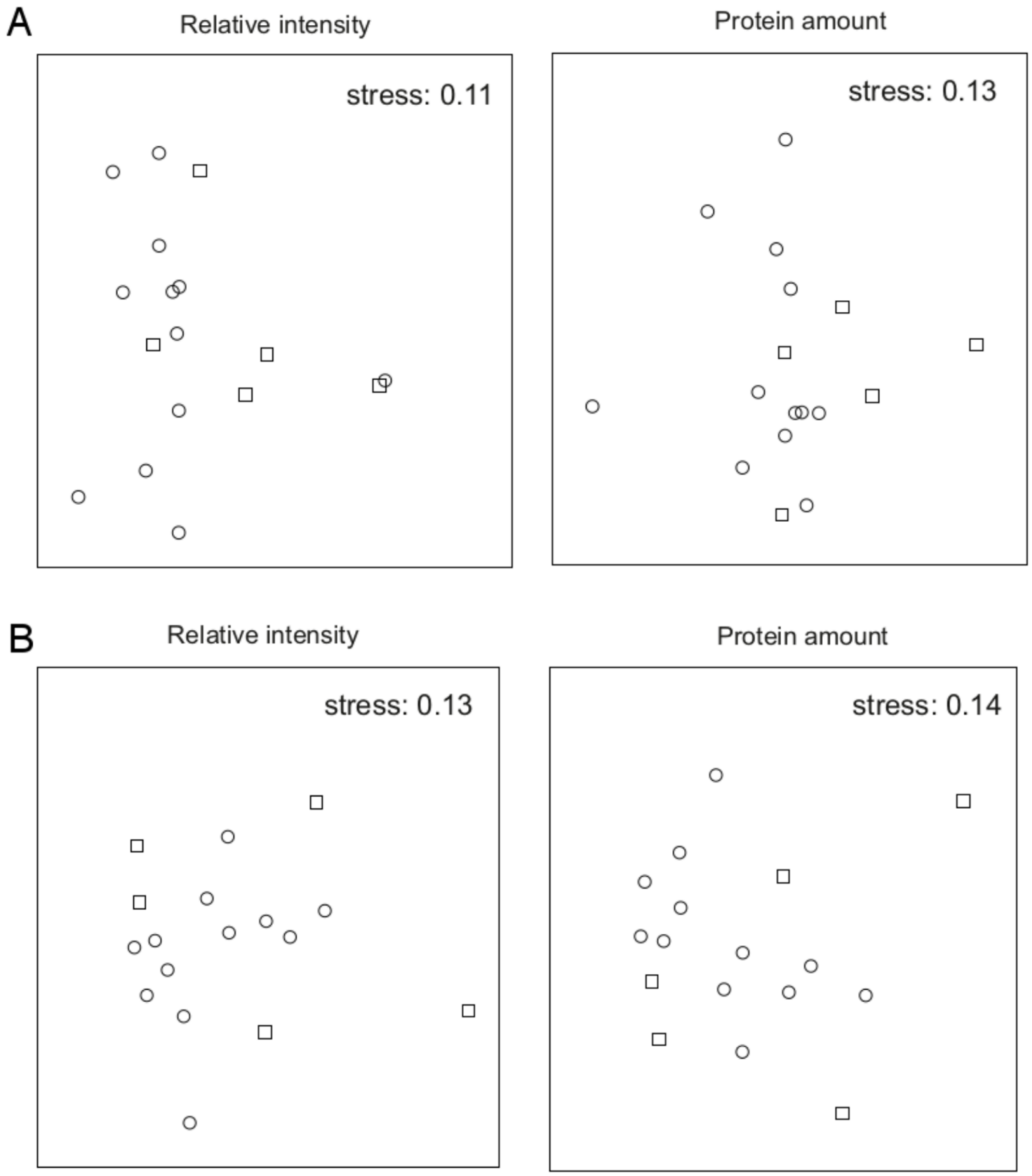

S1 Fig 8. Effects of seminatural conditions on the similarity of urinary protein profile fold-changes within males.

Protein profile similarity of dominant (circles) and subordinate (squares) males during the enclosure phase visualized using nonmetric multidimensional scaling. Data points closer together represent individuals with relatively high chemical similarity in fold change of relative intensity (left side) or fold change of protein amount (right side) of (A) MUPs and (B) non-MUP urinary proteins.

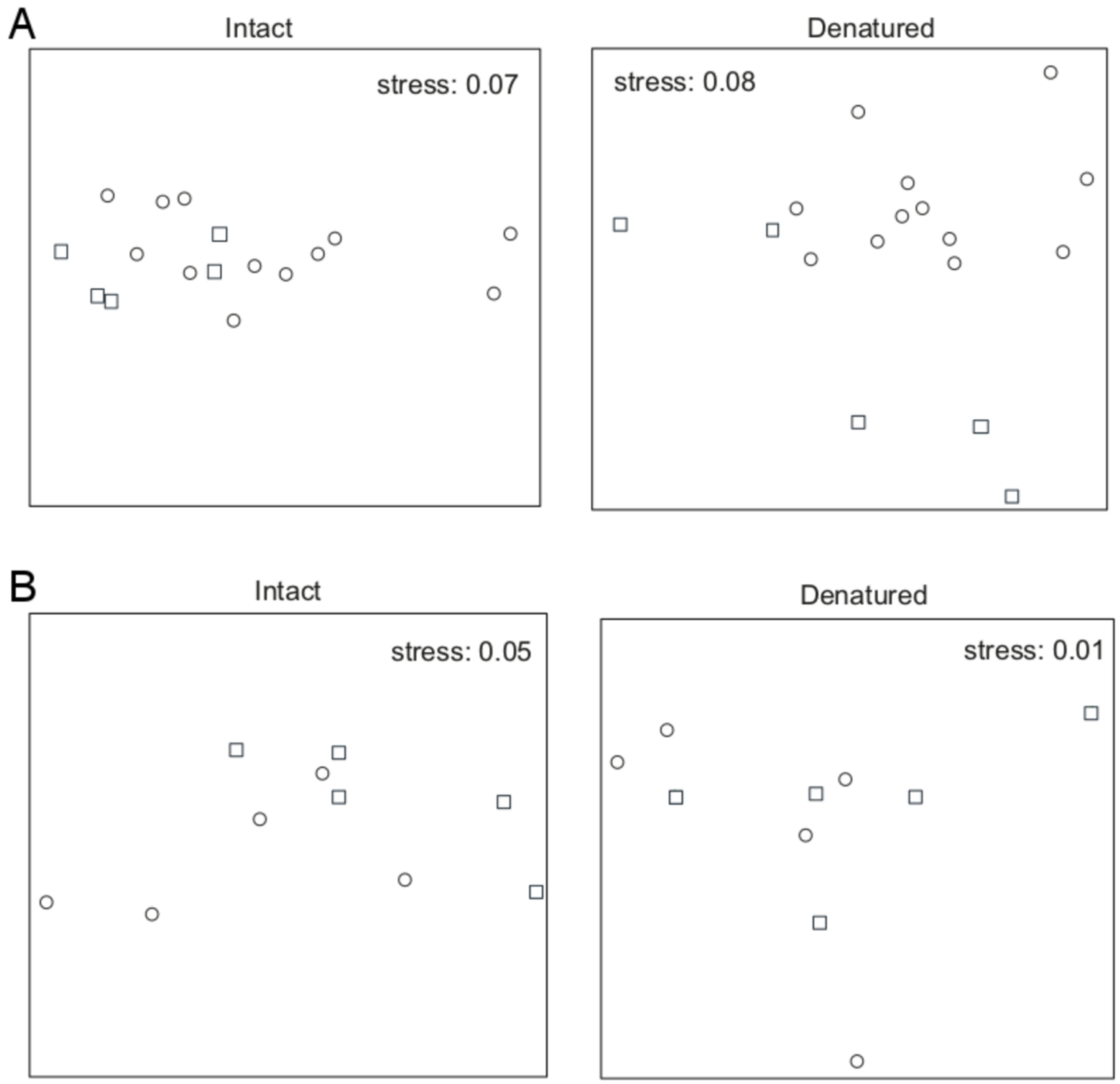

S1 Fig 9. Targeted volatile profile similarity between social status groups during seminatural conditions.

Volatile organic compound profile similarity based on 34 urinary compounds (targeted approach) of (A) dominant (circles) and subordinate (squares) males and (B) dominant (circles) and subordinate (squares) females during the enclosure phase visualized using nonmetric multidimensional scaling. Data points closer together represent individuals with relatively high chemical similarity in intact (left side) or denatured (right side) urine.

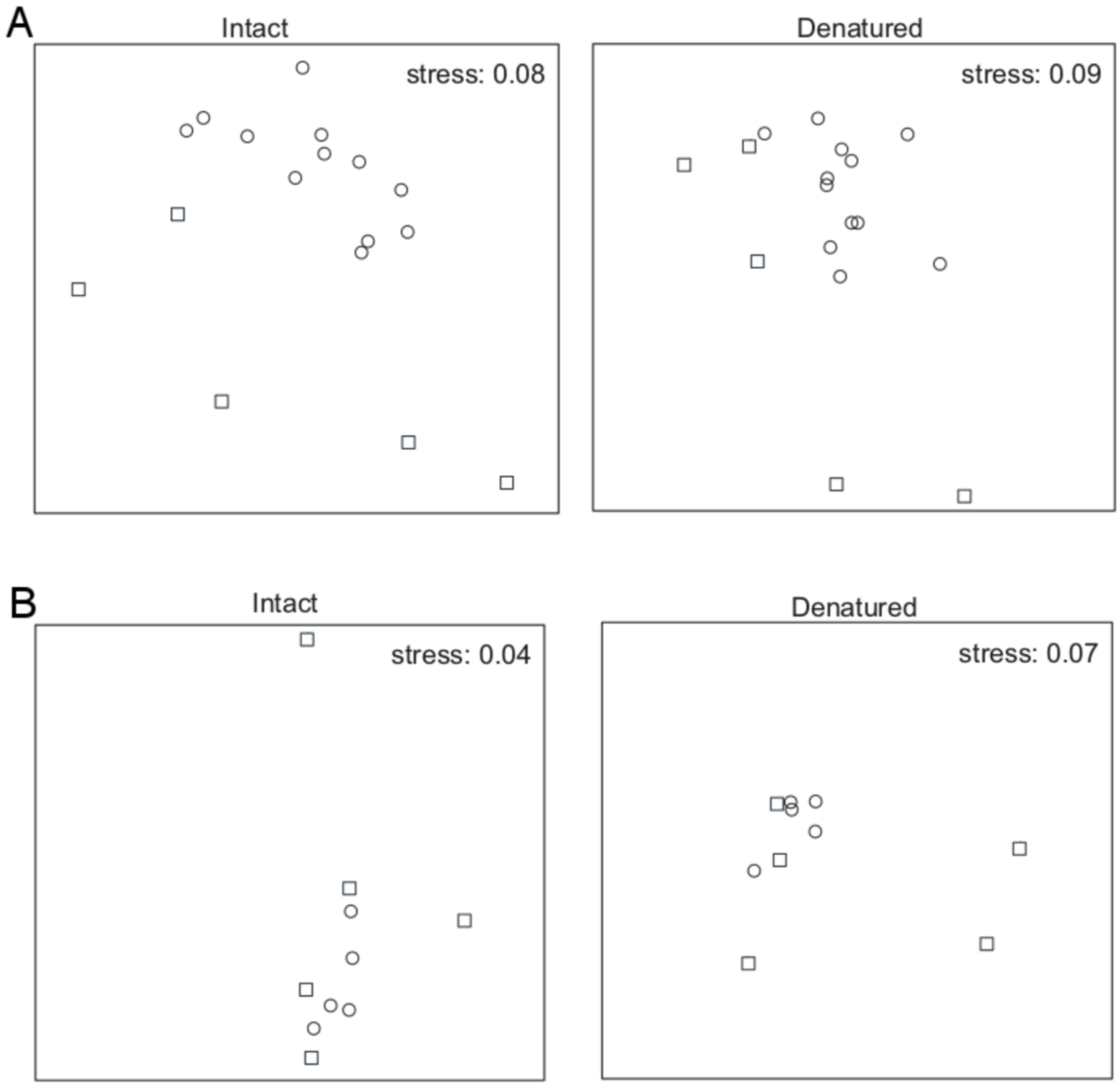

S1 Fig 10. Exploratory volatile profile similarity between social status groups during seminatural conditions.

Volatile organic compound profile similarity based on 962 GCMS peaks (exploratory data analysis) of (A) dominant (circles) and subordinate (squares) males and (B) dominant (circles) and subordinate (squares) females during the enclosure phase visualized using nonmetric multidimensional scaling. Data points closer together represent individuals with relatively high chemical similarity in intact (left side) or denatured (right side) urine.

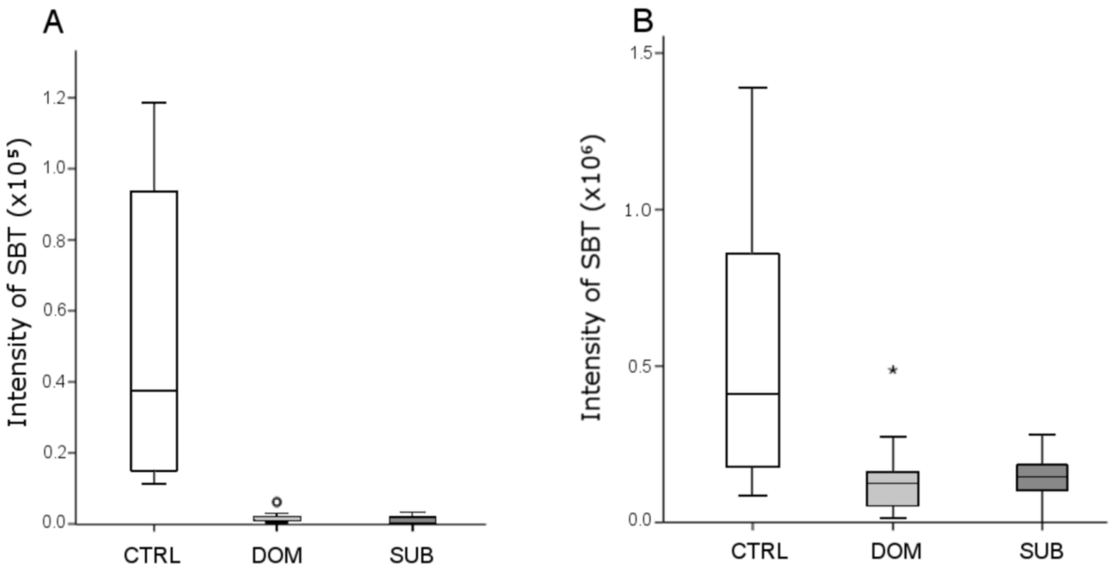

S1 Fig 11. SBT expression upregulated in caged control male urine.

Intensity of 2-sec-Butyl-4,5-dihydrothiazole (SBT) measured by gas chromatography-mass spectrometry in (A) intact and (B) denatured male urine samples of caged controls (white box), dominants (light gray box) and subordinates (dark gray box). Note the different y-axes scale between panels.

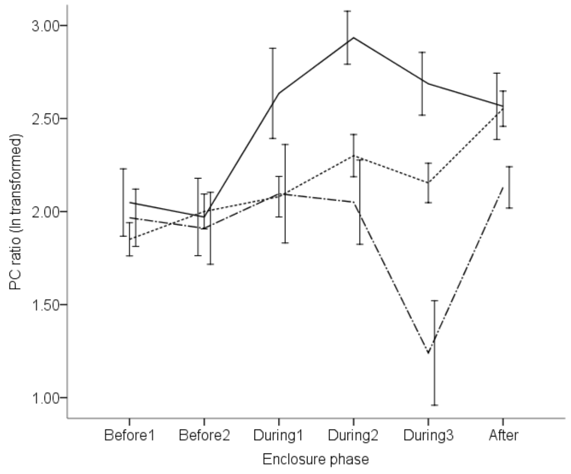

S1 Fig 12. Male urinary protein excretion (PC ratio) at each collection period.

Urinary protein excretion before, during, and after housing in seminatural enclosures for socially dominant (solid line) and subordinate (dot-dashed line) males compared to caged control males (dashed line). Mice were repeatedly sampled before (two collections) and during (three urine collections) the enclosure phases. A marked decrease for subordinates at the During3 collection was not significant (Tukey *post hoc*: During1_{SUB} v. During3_{SUB} $p = 0.73$; During2_{SUB} v. During3_{SUB} $p = 0.66$). Error bars indicate mean ± 1 s.e.m.
